# Modification of B-Nor Steroids Mediated by Filamentous Fungus *Fusarium culmorum*: Focus on 15α-Hydroxylase Activity

**DOI:** 10.3390/ijms252211913

**Published:** 2024-11-06

**Authors:** Alina Świzdor, Tomasz Janeczko, Anna Panek

**Affiliations:** Department of Food Chemistry and Biocatalysis, Wrocław University of Environmental and Life Sciences, Norwida 25, 50-375 Wrocław, Poland; tomasz.janeczko@upwr.edu.pl

**Keywords:** biotransformation, regioselective hydroxylations, B-nor steroids, *Fusarium culmorum*, steroidal 15α-hydroxylase

## Abstract

The metabolic activities of microorganisms to modify the chemical structures of organic compounds are an effective tool for the production of high-value steroidal drugs or active pharmaceutical ingredients (APIs). The integration of biotransformation into the synthesis of APIs can greatly reduce the number of reaction steps and achieve higher process efficiency, thus enabling their greener production. The current research efforts are focused on either the optimization of existing processes or identification of new potentially useful bioconversions. This study aimed to assess the catalytic abilities of the filamentous fungus *Fusarium culmorum* AM 282 to transform B-nor analogues (5(6→7)abeo compounds) of steroid hormones: androstenedione (AD), dehydroepiandrosterone (DHEA) and its acetate. Our previous studies have demonstrated that this strain is an active hydroxylating catalyst for many steroidal compounds with diverse structures. The results presented in this work showed that the hydroxylation of B-nor steroids occurred with the regio- and stereoselectivity typical of this strain in relation to the corresponding natural hormones of the standard 6:6 A/B series. After the transformations of B-nor-DHEA and its acetate, 15α-hydroxy-B-nor-DHEA was obtained as the sole product of the reaction, while the transformation of the AD analogue resulted in a mixture of its 15α- and 6α-hydroxy derivatives. A detailed analysis of the transformation course indicated that all the obtained hydroxy derivatives could be the result of the activity of the same enzyme. The presented results may provide a basis for research aimed at understanding the molecular nature of cytochrome P-450 monooxygenase from *F. culmorum* AM 282 with its ability for 15α-hydroxylation of steroidal compounds. An analysis of the pharmacokinetic and pharmacodynamic properties of the obtained metabolites with cheminformatics tools suggests their potential biological activity.

## 1. Introduction

Steroids are a widespread group of natural organic compounds playing diverse biological functions crucial for metabolism. Their physiological and pharmacological activity depends on the structure, primarily the location and stereochemistry of the functional groups attached to the steroid nucleus [1]. For instance, the introduction of the 11α-, 11β- or 16α-hydroxyl group and 9α-halogen atom into the steroid skeleton is utilized in the global production of corticosteroids. The 7-hydroxy derivatives of steroidal 5-en olefins exhibit several-fold higher immunoprotective and immunoregulatory properties than their parent compounds, but only 7α-hydroxylation of cholesterol is essential for the manufacture of bile acids [2]. Moreover, the introduction of scaffold-containing heteroatoms into steroid compounds represents a useful strategy for obtaining new anti-cancer drugs [3].

Chemical methods of hydroxylation often suffer from undesired byproducts of reactions or lack of necessary regio- or stereospecificity. A noteworthy technique solving these problems can be biotransformation using hydroxylating strains of fungi [4,5,6]. Although microbial catalysis is useful in the functionalization of steroids, especially in reactions taking place at the non-activated site of the molecule, it also has limitations related mainly to relatively low productivity (caused, for example, by low solubility of steroids in an aqueous environment and their high cellular toxicity) or/and selectivity of the processes. Most of the currently applied industrial microbial strains have been improved through technologies based on the selection processes and physicochemical mutagenesis [7]. The use of many currently available methodologies, including metagenomic approaches, genetic and protein engineering techniques, together with detailed studies of steroid metabolism pathways in bioconversion processes, may, in the future, lead to the development of bioprocesses for the industrial production of desired steroid molecules or steroid active pharmaceutical ingredients (APIs). Recently, steroid hydroxylase from the fungus *Thanatephorus cucumeris* was functionally reconstituted in *Mycolicibacterium neoaurum* for 15α-hydroxylation of progesterone [8]. A little earlier, the 13-ethyl-estr-4-ene-3,17-dione 15α-hydroxylase gene from *Penicillium raistricki* was characterized, cloned and heterologously expressed in *Saccharomyces cerevisiae* [9]. Similarly, the 11β-hydroxylation system from *Absidia orchidis* was characterized and expressed in *S. cerevisiae* for hydrocortisone biosynthesis [10]. Significant progress has also been made for the identification of C-14 hydroxylase from *Cochliobolus lunatus* [11], 19-hydroxylase from *Thanatephorus cucumeris* [12] and a new hydroxylase from *Nigrospora sphaerica* [13]. On the other hand, the study of the catalytic abilities of known microbial systems in the synthesis of new steroid compounds in order to assess their pharmacological usefulness is still in the current research trend [14,15,16,17,18].

Our previous works showed that the *Fusarium culmorum* AM 282 strain is an effective hydroxylating catalyst for a wide range of steroidal compounds with different structures [2,19,20,21,22,23]. This strain was also useful in the preparation of chiral (hydroxylated) derivatives of bi- and tricyclic enones (hexahydronaphthalenones) [24] and in the enantioselective reduction of prochiral ketones (tetralones) [25]. The high regio- and stereoselectivity of 15α- and 6β-hydroxylation of a series of 3-oxo-4-ene androstanes [19,21] and 7α-hydroxylation of 3β-hydroxy-5-ene steroids [20] prompted us to study the catalytic ability of this strain against B-nor androstanes. For our research, we chose B-nor analogues of steroidal hormones: androstenedione (AD), dehydroepiandrosterone (DHEA), and its acetate. To the best of our knowledge, this is the first report of the use of the *F. culmorum* strain in the biotransformation of B-nor steroids (5(6→7)abeo compounds).

B-nor steroids—compounds with an unusual skeleton of fused rings [6-5-6-5]—were the subject of scientific interest in both their synthesis and biological activity during the mid-1960s [26,27,28]. Numerous studies on the synthesis of this class of compounds have shown that the regio- and stereochemistry of many of their reactions differs from that typical of compounds belonging to the series of six-membered steroids [26]. It has been proven that the hormonal activity of B-nor-testosterone or B-nor-DHEA (B-nor-dehydroepiandrosterone) is significantly lower than their natural counterparts; hence, these compounds are considered antiandrogens [27]. It has also been indicated that 11-oxidized B-nor steroids may affect the central nervous system as therapeutic agents with antidepressant or sedative activity [28].

Renewed interest in the chemistry and biochemistry of B-nor steroids was prompted by the discovery of the apoptotic activity of marine B-norsterole–orostanal and the strong anti-tubercular effect of parguesteroles [29,30]. Then, it was shown via in vivo studies that structurally different synthetic abeo 5(6→7) cholestanes are highly active against *Mycobacterium tuberculosis*, while their natural six-membered ring counterparts remain inactive [31]. The results of bioactivity assays have shown that other synthetic B-nor steroids with a cholesteric side chain and 6-hydroxy-, 6-hydroxyimino-, 6-alkylthiosemicarbazone-, 6-cyano-group or 6-selenocyanate derivatives displayed an excellent antiproliferative activity against some cancer cells [32,33,34]. In other studies, it was found that the modification of the steroid backbone associated with contraction of the B ring slightly increased the activity of such molecules at the GABA_A_ receptor. B-nor analogues of allopregnanolone and pregnanolone, but not their derivatives with electronegative substituents (epoxide or ketone) in the B-ring, had a positive allosteric modulatory effect on GABA_A_ and, similarly to endogenous neurosteroids, they did not induce neurotoxicity [35]. Neuroactive steroids can be useful as sedatives, anesthetics, antistress, anticonvulsants or anxiolytics. Overall however, the precise role of B-nor steroids in biological processes remains less examined than their non-B-nor counterparts.

In the light of the above, it was reasonable to assess the physicochemical and pharmacokinetic properties of the obtained metabolites of the tested B-nor substrates. For this purpose, we used publicly available free to scientific community machine-learning-based tools—SwissADME (http://swissadme.ch, accessed on 22 February 2024) and Way2Drug PASS Online (htpps://www.way2drug.com/passonline/predict.php, accessed on 22 February 2024). The results obtained by computational methods are important for the future success of drug candidates and should be considered at the early stages of drug development. For example, an early ADME assessment in drug discovery has been shown to drastically reduce the fraction of pharmacokinetic failures in clinical phases [36], and the exploration of chemical libraries containing diverse structures of unexplored compounds by computer programs increases the chances of identifying pharmaceuticals interacting with multiple molecular targets resulting in additive or synergistic pharmacological effects [37].

## 2. Results and Discussion

In our previous studies on biotransformations by *F. culmorum* AM 282, we tested several steroids from the 4-en-3-oxo and 3β-hydroxy-5-en series of androstanes and pregnanes [19,20,21,22,23]. Those works demonstrated the ability of this microorganism to carry out regio- and stereospecific hydroxylation and that the position of the introduced hydroxyl group depended on the structure of the substrate. The hydroxylation occurred at the equatorial position of the steroid skeleton (15α- or rarely 12β-) whereas the axial hydroxyl group was introduced mainly into the allylic position (6β- or 7α-). For example, the 5-ene substrate DHEA was hydroxylated entirely at the 7α-position [20] while the hydroxylation at 15α occurred in the presence of the 4-ene-3-oxo system. Only the compounds with the oxygen function at C-17 (ketone or β-alcohol) were also hydroxylated at the 6β-position. Moreover, ketone–alcohol interconversion at C-17 for the substrates as well as their products of hydroxylation was detected [19]. A detailed analysis of the transformations course suggested that all obtained hydroxy derivatives might result from the activity of the same enzyme. Fungal enzymatic hydroxylation reactions are most commonly mediated by cytochrome P450 systems consisting of a monooxygenase CYP containing a heme-iron center and a NADPH-cytochrome P450 reductase [8,38].

**Figure 1:**
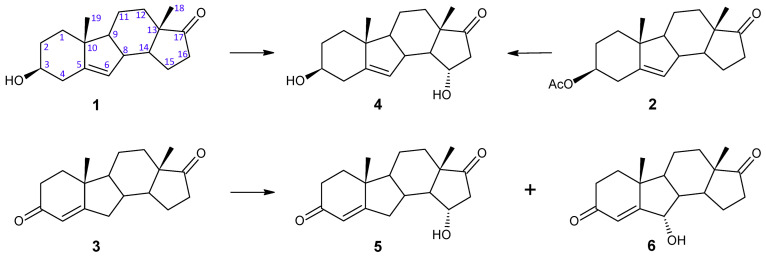
Transformation of B-nor analogues of natural steroidal hormones by *F. culmorum* AM 282.

The study presented in this work showed that hydroxy derivatives (but not 17β-hydroxy compounds, which are products of steroidal 17β-dehydrogenase) were also obtained after the conversion of B-nor analogues of natural steroid hormones DHEA and AD (Figure 1). B-nor-DHEA (**1**) was prepared from B-nor-DHEA acetate (**2**) by its base hydrolysis. B-nor-AD (**3**) was synthesized by Oppenauer oxidation of **1**. The structures of the substrates and obtained products were established by using spectroscopic techniques (^1^H NMR and ^13^C-NMR) (Supplementary Materials Figures S1–S8).

After a 24 h transformation of 3β-hydroxy-B-norandrost-5-en-17-one (B-nor-DHEA) (**1**), only one product was identified (99% according to GC analysis) in the post-reaction media extracts. The NMR spectra of this metabolite showed a new multiplet at δ_H_ 4.31–4.35 ppm and δ_C_ 70.5 ppm, which suggested hydroxylation at an equatorial position of the steroid molecule. The lack of changes in chemical shifts of the C-18 and C-19 methyl signals and the presence of the signal of 16β-proton at δ_H_ 2.90 ppm (dd, *J* = 7.7, 19.1 Hz) were in agreement with those reported in the literature for 15α-hydroxylation [39]. This was further supported by the ^13^C NMR, which showed downfield shifts for β-carbons C-16 (Δ 9.1 ppm) and C-14 (Δ 7.2 ppm). Based on the above data, the structure of **4** has been established as 3β,15α-dihydroxy-B-norandrost-5-en-17-one (Supplementary Materials Figures S9–S12). The same product (identified on the basis of *R_t_* from GC and *R_f_* from TLC) but with lower yield (76% according to GC analysis) was obtained from 3β-acetoxy-B-norandrost-5-en-17-one (**2**) (Figure 1).

Time course experiments evidently indicated (Supplementary Materials Table S1) that the first stage of the reaction of **2** was hydrolysis of the ester bond because 15α-hydroxy-B-nor-DHEA acetate was not identified in any of the extracts. Additionally, the control experiment with the suspension of the autoclaved mycelium of *F. culmorum* did not give any transformation product and confirmed that **2** is stable under the incubation conditions. Hydrolysis of the ester bond was also the first step in the biotransformation of esters of the natural steroid series [22]. The 15α-hydroxy-B-nor-DHEA (**4**) was previously obtained in ca. 15% yield from an analog of B-nor-cortexolone after its hydroxylation by *Beauveria bassiana* and then oxidation with sodium bismuthate. The analog of B-nor-cortexolone was converted to the 15α-hydroxy derivative as one of three products of this catalysis [40].

**Figure 2:**
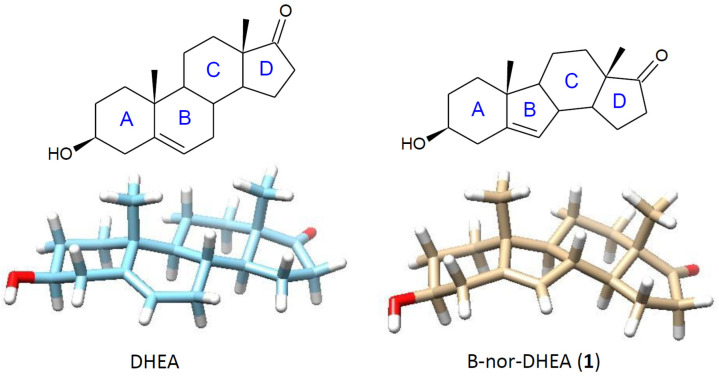
Structures and spatial models of DHEA and B-nor-DHEA (**1**).

The effective and regioselective 15α-hydroxylation of the B-nor derivative of DHEA (**1**) by *F. culmorum* could be predicted based on the results of transformation of its natural analogue (DHEA) and comparison of the spatial models of both substrates (Figure 2). Assuming that the 7α-hydroxylation of DHEA and 15α-hydroxylation of B-nor-DHEA (**1**) are conducted by the same enzyme and the active site of this enzyme is located between the C-7 and C-15 atoms of the steroid system, the 15α-H atom of **1** should be closer to the heme oxygen of cytochrome P-450 hydroxylase than the 7α-H atom of DHEA.

**Figure 3:**
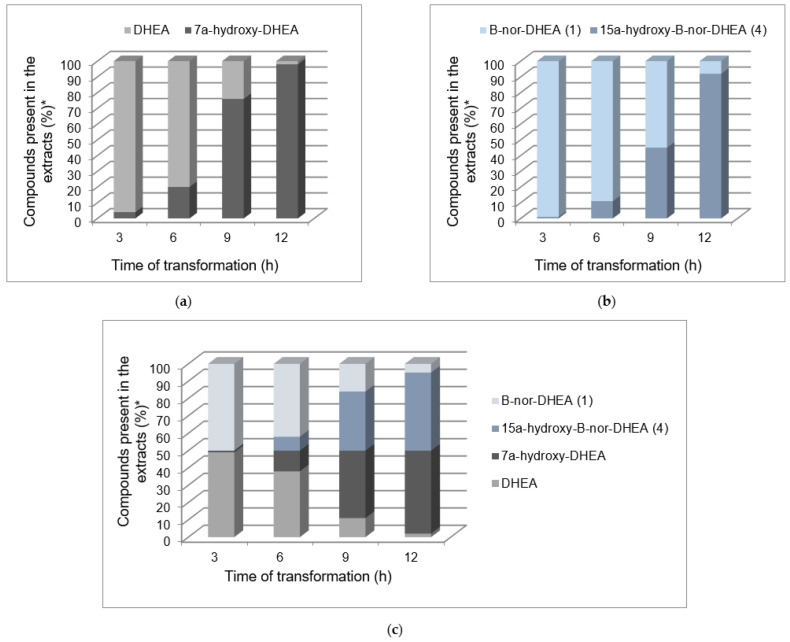
The composition of extracts obtained after transformation of (**a**) DHEA, (**b**) B-nor-DHEA (**1**) and (**c**) an equimolar mixture of DHEA and B-nor-DHEA (**1**). * Determined by GC analysis. The presented values are the average of three independent experiments and relative differences between the border values have not exceeded 5%. Exact mean values and standard deviations are provided in Supplementary Materials—Table S2.

To test this hypothesis, we performed simultaneous transformations of DHEA, its analog **1** and an equimolar mixture of both substrates. The results are presented on the graphs (Figure 3). A comparison of the percentage of metabolites of both substrates in the extracts obtained after the third and subsequent hours of transformation indicates that the reaction rate increases significantly over time. The reaction mixture after 3 h of DHEA incubation contained 4% of 7α-hydroxy-DHEA; after another 3 h its content increased to 20%; after another three hours it reached 76%; and finally, after another 3 h, almost complete substrate conversion was noted. A similar conversion profile in 3-h time intervals was observed in the transformation of B-nor-DHEA (**1**). The content of 15α-hydroxy-DHEA (**4**) increased from 1% to 11%, 45% and, finally, 92%, respectively. These results suggest that both substrates are inducers of the enzyme(s) catalyzing their hydroxylation.

**Figure 4:**
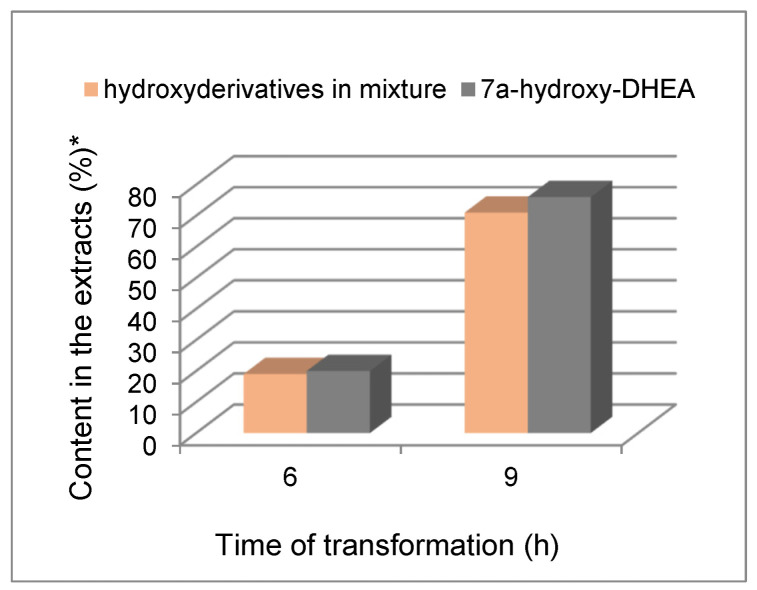
Overall catalytic effect of the transformation of DHEA and an equimolar mixture of DHEA and B-nor-DHEA (**1**). * Determined by GC analysis. The presented values are the average of three independent experiments and relative differences between the border values have not exceeded 5%. Exact mean values and standard deviations are provided in Supplementary Materials—Table S2.

If the 15α-hydroxylation of **1** and 7α-hydroxylation of DHEA was the result of the action of different enzymes, a change in the reaction selectivity of simultaneously transformed substrates at an equimolar mixture would be expected. At the same time, the rate of transformation of each of the components of the mixture would be comparable to the rate of transformation appropriate for each of the tested substrates. In a mixture composed of both substrates, the presence of DHEA accelerated the hydroxylation reaction of its B-nor analog. Between 6 h and 9 h of transformation of **1** in the mixture of both substrates, its conversion was higher by about 20%. Also, the selectivity of the hydroxylation remained unchanged. In addition, the overall catalytic effect of the transformation in the mixture of both substrates was comparable to the efficiency of DHEA catalysis (Figure 4).

The incubation of B-nor-AD (**3**) with *F. culmorum* resulted in the isolation of unreacted starting material (14% according to GC analysis) and a mixture of two metabolites (Figure 1). Their content in the post-reaction mixture was comparable. The first product had new resonances at δ_H_ 4.32 ppm and δ_C_ 70.4 ppm. The multiplicity of this signal, the lack of significant shifts in the methyl group signals in the ^1^H NMR spectrum and the lack of changes in the chemical shifts of protons and carbon atoms of the A-C rings suggested that the OH group was introduced into the D ring at the equatorial 15α-position [39]. Downfield shifts of β-carbon signals in the ^13^C NMR for C-14 (Δ 7 ppm) and C-16 (Δ 9.8 ppm) confirmed hydroxylation at C-15. All these results fully confirmed the structure of **5** as 15α-hydroxy-B-norandrost-4-en-3,17-dione (15α-hydroxy-B-nor-androstenedione) (Supplementary Materials Figures S13–S16). Metabolite **5** is a new compound, but 15α-hydroxy-B-norcortexolone, from which 15α-hydroxy-B-nor-androstenedione can be derived, was one of the four metabolic products of B-nor-cortexolone transformation by *Beauveria bassiana*. It was obtained with ca. 4% yield [40].

The NMR spectra of compound **6** showed a new downfield signal for the oxygen-bearing methine proton at δ_H_ 4.68 ppm (dd, *J* = 1.0; 7.0 Hz) and δ_C_ 71.9 ppm, which indicated the introduction of a hydroxyl group into the molecule. A downfield shift of the vinyl H-4 signal (Δ 0.23 ppm) in ^1^H NMR spectrum suggested hydroxylation in close proximity to the olefin moiety. Downfield shifts of H-9, H-14 and H-15 signals indicated 6α-hydroxylation. Further evidence for this hydroxylation was provided by the downfield β-carbon shift of C-8 (Δ 5.3 ppm) and γ-carbon upfield shifts of C-9 (Δ 4.2 ppm) and C-14 (Δ 5.2 ppm). The α-stereochemistry of the OH group at C-6 was finally confirmed by the NOESY spectrum, which showed correlations between 6β-H and 8-H (δ_H_ 2.06–2.16 ppm), 15β-H (δ_H_ 1.74–1.75 ppm) and C-19 methyl group (δ_H_ 1.14 ppm) signals. These data led to the identification of **6** as 6α-hydroxy-B-norandrost-4-en-3,17-dione (6α-hydroxy-B-nor-androstenedione (Supplementary Materials Figures S17–S21). Compound **6** was previously obtained in ca. 6% yield from B-nor-cortexolone after its hydroxylation by *Beauveria bassiana* and then oxidation with sodium bismuthate. B-nor-cortexolone was converted to the 6α-hydroxy derivative as one of four products of this catalysis [40]. It was also one of three metabolites (yield 0.8%) obtained after the transformation of B-norandrostenedione by *Rhizopus arrhizus* ATCC 11145 [41].

**Figure 5:**
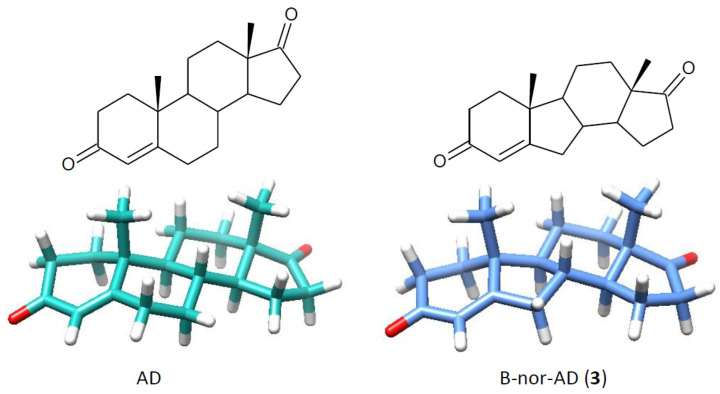
Structures and spatial models of AD and B-nor-AD (**3**).

The regioselectivity of hydroxylation B-nor-AD (**3**) and its natural analog (AD) by *F. culmorum* show some similarities—15α-hydroxylation took place in both substrates, but the configuration of the hydroxyl group introduced at the C-6 position was different (6β in AD and 6α in B-nor-AD). Assuming that both hydroxylations take place in the same substrate–enzyme complex, the first question arises why the analogically located (relative to H_6α_ of **3**) H_7α_ of AD was not oxidized and 7α-hydroxy derivative was not obtained from AD, while its B-nor analog (**3**) produces 50% of the 6α-alcohol. An analysis of the spatial models of both substrates indicates that the distances of H_7α_ in AD and H_6α_ in B-nor-AD from the hypothetical active site of the enzyme are comparable. The observed difference in the regioselectivity of hydroxylation could be the result of a different orientation of these bonds. The C_7_-H_7α_ bond in the AD molecule faces away from the oxygen atom of the enzyme’s heme system, and the C_6_-H_6α_ bond in B-nor-AD faces this atom (Figure 5).

Additionally, the hydroxylation at C_6_ is favored by its allyl character. It seems that the selectivity of the allylic hydroxylation of B-nor-AD depends largely on its interaction with the enzyme and is less subject to the stereoelectronically controlled axial addition of electrophilic oxygen to the enol system from 4-ene-3-ketone. Despite the α and β positions at C-6 being stereochemically equivalent with respect to the plane of the O-C3-C4-C5 enone system, formation of the 6β-alcohol was not observed.

The 15α-Hydroxylated steroids serve as key intermediates in the production of gestodene [4], which is a major component of third-generation contraceptives [9]. The presence of a hydroxyl group in this position allows 15(16)-dehydratation, and the presence of a double bond at C-15 may increase the antiviral activity of the steroids. In vitro studies have established that 15(16)-dehydroderivatives of epiandrosterone have ability to inhibit the replication of arenaviruses, e.g., the Junin virus causing hemorrhagic fever. These derivatives were also active against the vesicular stomatitis virus [42].

**Figure 6:**
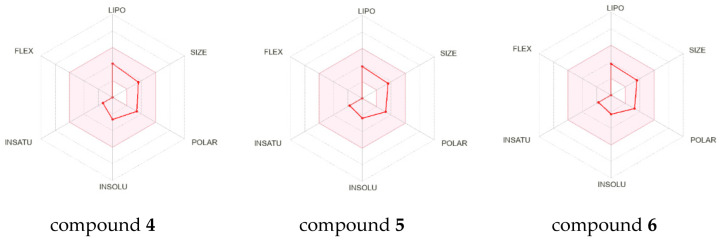
The bioavailability radars of metabolites **4**–**6**. The pink area represents the optimal range for lipophilicity (XLOGP3 between −0.7 and +5), size (MW between 150 and 500 g/mol), polarity (TPSA between 20 and 130 Å^2^), insolubility (Log S ESOL between −6 and 0), insaturation (fraction Csp^3^ between 0.25 and 1) and flexibility (rotatable bonds between 0 and 9).

In an effort to assess the potential of the physicochemical properties, pharmacokinetics, drug-likeness and medicinal chemistry friendliness of the obtained hydroxylated B-nor derivatives of DHEA and AD, we applied the online tool SwissADME (http://swissadme.ch, accessed on 22 February 2024), developed and managed by the Molecular Modeling Group of the Swiss Institute of Bioinformatics (https://www.sib.swiss/, accessed on 22 February 2024) [43], and the Way2Drug PASS Online protocols (htpps://www.way2drug.com/passonline/predict.php, accessed on 22 February 2024). According to the Swiss-ADME analysis, all parameters of compounds **4**–**6** (lipophilicity, size, polarity, insolubility, insaturation and flexibility) were within the optimal range, indicating the suitability of these structures as potential drugs (Figure 6). These were in agreement with all five drug-likeness sets of criteria: Lipinski, Ghose, Veber, Egan and Muegge. The introduction of 15α- or 6α-hydroxyl group to the substrates significantly increased their water solubility (from 0.187 mg/mL for compound **1** to 0.643 mg/mL for compound **4** and from 0.327 mg/mL for compound **3** to 1.14 mg/mL for compound **5** and 1.38 mg/mL for **6**) and decreased their lipophilicity (from Log P_o/w_ 3.11 for **1** to 2.30 for **4** and from Log P_o/w_ 3.14 for **3** to 2.33 for **5** and 2.31 for **6**). In addition, the BOILED-Egg model was analyzed to provide insight into the possibility of gastrointestinal absorption and brain penetration [44]. According to the results from this model, all three tested metabolites (similar to the parent compounds) showed high gastrointestinal absorption and could passively penetrate the blood/brain barrier, but only new metabolites **4**–**6** could be removed from the brain by the P-glycoprotein. Based on ADME analyses, none of the obtained derivatives should be an inhibitor of key cytochromes P450 (CYP1A2, CYP2C19, CYP2C9, CYP2D6, CYP3A4), which are involved in the metabolism of xenobiotics. More detailed insight into the bioavailability and characteristics of the tested compounds can be found in the Supplementary Materials (Figures S22–S23).

In silico studies using the Way2Drug PASS Online platform showed that the substrates and products of their transformation should be, with very high probability, substrates for many cytochrome P-450 monooxygenases (Table 1).

The obtained products as well as their parent compounds should be substrates for UDP-glucuronosyltransferase and sulfotransferase (Table 2)—enzymes which are involved in the metabolism, detoxification and removal of xenobiotic chemicals (environmental, pharmaceutical), most drugs and toxins from the body. The transfer of a sulfonate group from a donor substrate to an acceptor substrate (drug, hormone, neurotransmitter) with a hydroxy or amine group is important for the metabolism and regulation of steroids in various physiological processes [45,46]. UDP-glucuronosyltransferases are responsible for the processes of glucuronidation, which is a major part of the phase II metabolic conjugative pathway of xenobiotics [47]. The increased solubility of glucuronide allows it to be eliminated from the body through the kidneys.

PASS simulations also showed a high probability of limitation of morphine 6-dehydrogenase activity by obtained B-nor metabolites. The highest probability of such activity was assigned to 15α-hydroxy-B-nor-AD (**5**) (Table 2). The inhibition of morphine 6-dehydrogenase, an enzyme that catalyzes morphinone production from morphine, prevents the development of tolerance to morphine and weakens physical dependence and withdrawal behaviors [48].

The tested compounds may have some lysase inhibitory activity (Table 2). This is particularly important for steroidal C_17,20_-lyase. The inhibition of this enzyme decreases the synthesis of the testicular androgens, as well as adrenal androgens, and it is a promising strategy for the treatment of prostate cancer [49].

Based on prediction studies using the Way2Drug PASS Online platform, the described compounds with high probability should be inhibitors of prostaglandin-E2 9-reductase (PGE_2_) and 27-hydroxycholesterol 7α-monooxygenase (Table 2). The activity of prostaglandin E2 9-reductase reversibly changes PGE_2_ into PG_F2α_ and may be responsible for the control of prostaglandin levels in the body [50]. The 27-hydroxycholesterol 7α-monooxygenase is involved in an alternative bile acid biosynthesis pathway [51]. According to the PASS analyses, the described compounds should very likely be the inhibitors of testosterone 17β-dehydrogenase (Table 2). This is an enzyme that catalyzes the reduction of 17-keto- or the oxidation of 17β-hydroxysteroids using NADP^+^ as cofactor, playing an important role in the regulation of steroid hormones in the body. The 17β-HSD inhibitors are therefore useful tools for elucidating the role of these enzymes in specific biological systems or for therapeutic purposes, where they block the synthesis of active hydroxysteroids stimulating hormone-dependent diseases such as breast and ovarian cancers, as well as prostate cancer, benign prostatic hyperplasia or hirsutism [52].

The obtained derivatives of B-nor steroids (especially 15α-hydroxy) should also, with a high probability, find potential use as respiratory analeptics, ovulation inhibitors or cholesterol antagonists. They should also have some antihypercholesterolemic activity, stimulate protein synthesis and be useful in the treatment of menopausal disorders (Table 3).

## 3. Materials and Methods

### 3.1. Chemicals

The 3β-Hydroxy-B-norandrost-5-en-17-one (**1**) was prepared by base-catalyzed hydrolysis of 3β-acetoxy-B-norandrost-5-en-17-one (**2**) and was found to be in excess of 99% purity following GC analysis. Compound **2** was obtained from the resources of the Department of Chemistry, Wrocław University of Environmental and Life Sciences (Poland). The B-norandrost-4-en-3,17-dione (**3**) was converted by Oppenauer oxidation of 3β-hydroxy-B-norandrost-5-en-17-one (**1**) and was found to be in excess of 97% purity according to GC analysis. Dehydroepiandrosterone (3β-hydroxyandrost-5-en-17-one, DHEA) was purchased from Biosynth Carbosynth^®^ (Berkshire, UK). The 7α-Hydroxy-DHEA was prepared in our previous manuscript [53].

### 3.2. Preparation of 3β-Hydroxy-B-norandrost-5-en-17-one (**1**)

To 480 mg (1.6 mmol) of 3β-acetoxy-B-norandrost-5-en-17-one (**2**) in 20 mL of MeOH, 480 mg of Na_2_CO_3_ (4.7 mmol) in 2.5 mL of water was added, and the solution was stirred at room temperature (RT) for 2 h. After evaporation of the methanol under reduced pressure, the residue was diluted with water, and the resulting crude product was extracted with diethyl ether. The extract was dried over MgSO_4_, and the organic solvent was again evaporated. Purification by column chromatography in hexane:acetone (2:1 *v*/*v*) as eluent gave 315 mg (72%) of 3β-hydroxy-B-norandrost-5-en-17-one (**1**).

**3β-Hydroxy-B-norandrost-5-en-17-one (1)**: ^1^H NMR (600 MHz, CD_3_OD) δ_H_: 0.92 (3H, s, 18-Me), 0.93 (3H, s, 19-Me), 1.11–1.14 (1H, m, 9-H), 1.14–1.19 (1H, m, 1α-H), 1.24 (1H, dt, *J* = 5.0, 12.4 Hz, 12α-H), 1.48–1.60 (4H, m, 2α-H, 11α-H, 11β-H, 14-H), 1.72–1.77 (1H, m, 15α-H), 1.78–1.84 (3H, m, 1β-H, 2β-H, 12β-H), 1.95–1.99 (1H, m, 16α-H), 1.99–2.04 (1H, m, 15β-H), 2.07–2.13 (1H, m, 4α-H), 2.42–2.47 (1H, m, 4β-H), 2.48–2.50 (1H, m, 8-H), 2.57 (1H, ddd, *J* = 1.97, 4.71, 13.67 Hz, 16β-H), 3.45 (1H, tt, *J* = 4.3, 11.2 Hz, 3α-H), 5.45 (1H, s, 6-H). ^13^C NMR (151 MHz, CD_3_OD) δ_C_: 14.5 (C-18), 15.6 (C-19), 21.2 (C-11), 23.3 (C-15), 32.6 (C-2), 33.1 (C-12), 36.6 (C-4), 37.3 (C-16), 38.3 (C-1),45.9 (C-10), 47.0 (C-8), 50.6 (C-13), 51.2 (C-14), 64.1 (C-9), 72.2 (C-3), 124.3 (C-6), 151.7 (C-5), 223.0 (C-17). The spectral data of this compound were in good agreement with that reported in the literature [41]. EI-MS m/z 274 [M]^+^ (65), 259 (60), 231 (49), 215 (58), 187 (59), 145 (56), 131 (68), 105 (80), 91 (100). See Figure S24 in the Supplementary Materials for the MS spectrum. Retention time: *R_t_* = 2.72 min.

### 3.3. Preparation of B-Norandrost-4-en-3,17-dione (**3**)

First, 200 mg (0.73 mmol) of 3β-hydroxy-B-norandrost-5-en-17-one (**1**) was dissolved in a mixture of dry toluene (35 mL) and cyclohexanone (8 mL). The reaction mixture was heated at reflux in anhydrous conditions (CaCl_2_), and then a solution of aluminum isopropoxide (280 mg) in dry toluene (1.5 mL) was added. After a 4 h reaction, the organic solvents were removed under reduced pressure, the remaining suspension acidified with 10% sulfuric acid and the product extracted with diethyl ether. The extract was washed with a solution of NaHCO_3_ and water, dried over MgSO_4_, and then the organic solvent was evaporated. Purification by column chromatography in hexane:acetone (2:1 *v*/*v*) as eluent gave 130 mg (66%) of B-norandrost-4-en-3,17-dione (**3**).

**B-norandrost-4-en-3,17-dione (3)**: ^1^H NMR (600 MHz, CD_3_OD) δ_H_: 0.96 (1H, s, 18-Me), 1.07–1.12 (1H, m, 9-H), 1.15 (1H, s, 19-Me), 1.27–1.32 (1H, m, 12α-H), 1.49–1.59 (2H, m, 11α-H, 14-H), 1.63–1.67 (1H, dm, *J* = 13.2 Hz, 11β-H), 1.71–1.77 (2H, m, 1α-H; 15β-H), 1.85 (1H, dm, *J* = 13.1 Hz, 12β-H), 1.95–1.99 (1H, m, 15α-H), 2.05–2.08 (1H, m, 1β-H), 2.10–2.18 (2H, m, 16α-H, 8-H), 2.25 (1H, ddd, *J* = 1.9, 10.1, 19.1 Hz, 6α-H), 2.33 (1H, dm, *J* = 18.7 Hz, 2α-H), 2.46 (1H, dd, *J* = 8.7 Hz, 19.5 Hz, 16β-H), 2.60–2.66 (1H, m, 2β-H), 2.82 (1H, ddd, *J* = 1.8, 7.9, 19.1 Hz, 6β-H), 5.80 (1H, br s, 4-H). ^13^C NMR (151 MHz, CD_3_OD) δ_C_: 14.6 (C-18), 17.5 (C-19), 21.0 (C-11), 23.3 (C-15), 32.5 (C-12), 34.4 (C-2), 34.5 (C-6), 36.2 (C-1), 36.6 (C-16), 39.3 (C-8), 45.2 (C-10), 50.5 (C-13), 52.2 (C-14), 59.6 (C-9), 123.1 (C-4), 182.0 (C-5), 202.1 (C-3), 222.7 (C-17). The spectral data of this compound were in good agreement with that reported in the literature [41]. EI-MS m/z 272 [M]^+^ (32), 257 (13), 244 (18), 230 (100), 216 (17), 159 (19), 145 (16), 131 (21), 105 (81), 91 (46). See Figure S25 in the Supplementary Materials for the MS spectrum. Retention time: *R_t_* = 3.71 min.

### 3.4. Microorganism

The fungal filamentous strain *Fusarium culmorum* AM282 used in this study was obtained from the collection of the Institute of Biology and Botany, Medical University of Wrocław. The fungus was maintained on Sabouraud 4% dextrose-agar slopes at 4 °C and freshly subcultured before use in the transformation experiments.

### 3.5. Conditions of Cultivation and Transformation

The general experimental and fermentation details have been described previously in our paper [54]. A substrate as a 5% solution in acetone was added to each of the cultures of microorganisms to give a final substrate concentration of 0.250 g L^−1^. The biotransformation continued as long as the substrate was metabolized. The progress of the reaction was monitored by TLC. Each experiment was performed in triplicate.

### 3.6. Isolation and Identification of Products

The general procedures of product isolation have been described in our previous paper [54]. The crude post-transformations extracts were analyzed by TLC or/and GC and then chromatographed on a column of silica gel (Silica gel 60 (0.040–0.063 mm)). The GC analyses were performed using Hewlett Packard 5890A Series II GC instrument (FID, carrier gas H_2_ at flow rate of 2 mL min^−1^, Hewlett Packard Company, Wilmington, DE, USA) with HP-5 column (cross-linked 5% Ph-Me-Silicone; 25 m × 0.32 mm × 0.52 µm film thickness). The applied temperature program was 220 °C/1 min gradient 4 °C/min to 245 °C, gradient 30 °C to 300 °C/1 min; the injector and detector temperature was 300 °C. The NMR spectra were recorded on a Bruker Avance^TM^ 600 MHz spectrometer (Bruker, Billerica, MA, USA) and measured in CD_3_OD. The characteristic shift values in the ^1^H NMR, ^13^C NMR and 2D HSQC NMR spectra in comparison with the starting compounds were used to determine the structures of the metabolites, in combination with DEPT analysis to identify the nature of the carbon atoms. The stereochemistry of the hydroxyl group was deduced on the basis of the NOESY experiment. The GC-MS analyses were performed on a GCMS-QP2010 apparatus (Shimadzu, Kyoto, Japan) with a ZB5 (crosslinked phenyl methyl siloxane) (30 m × 0.25 mm × 0.25 μm) capillary column (Phenomenex, Torrance, CA, USA). The following temperature program was used: 180 °C/1 min^−1^, gradient 5 °C min^−1^ to 300 °C/10 min^−1^. The injector temperature was 280 °C. Melting points were determined on a Boetius apparatus and are uncorrected. A flowchart covering the main stages of the study is placed in the Supplementary Materials (Scheme S1).

### 3.7. Products Isolated After Transformations

**3β,15α-Dihydroxy-B-norandrost-5-en-17-one (4)**: (76 mg, 89% mol.); mp 253 °C (from acetone) (lit. [40] 252–255 °C). ^1^H NMR (600 MHz, CD_3_OD) δ_H_: 0.94 (3H, s, 18-Me), 0.94 (3H, s, 19-Me), 1.10–1.14 (1H, m, 9-H), 1.14–1.19 (1H, m, 1α-H), 1.33 (1H, dt, *J* = 4.5, 12.7 Hz, 12α-H), 1.47–1.58 (4H, m, 2α-H, 11α-H, 11β-H, 14-H), 1.75–1.81 (3H, m, 1β-H, 2β-H, 12β-H), 1.95–2.00 (2H, m, 4α-H, 16α-H), 2.54–2.59 (2H, m, 4β-H, 8-H), 2.90 (1H, dd, *J* = 7.7, 19.1 Hz, 16β-H), 3.46 (1H, tt, *J* = 4.3; 11.2 Hz, 3α-H), 4.31–4.35 (1H, m, 15β-H), 5.69 (1H, s, 6-H). ^13^C NMR (151 MHz, CD_3_OD) δ_C_: 15.5 (C-19), 15.8 (C-18), 21.1 (C-11), 32.6 (C-2), 33.3 (C-12), 37.3 (C-4), 38.3 (C-1), 45.2 (C-10), 46.0 (C-8), 46.4 (C-16), 53.0 (C-13), 58.4 (C-14), 64.0 (C-9), 70.5 (C-15), 72.2 (C-3), 125.5 (C-6), 151.1 (C-5), 218.8 (C-17). EI-MS m/z 290 [M]^+^ (28), 275 (66), 257 (37), 244 (27), 231 (53), 218 (37), 185 (49), 145 (63), 131 (60), 105 (76), 91 (100). See Figure S26 in the Supplementary Materials for the MS spectrum. Retention time: *R_t_* = 4.33 min.

**15α-Hydroxy-B-norandrost-4-en-3,17-dione (5)**: (27 mg, 32% mol.); ^1^H NMR (600 MHz, CD_3_OD) δ_H_: 0.97 (3H, s, 18-Me), 1.05–1.09 (1H, m, 9-H), 1.15 (3H, s, 19-Me), 1.39 (1H, dt, *J* = 4.0, 12.8 Hz, 12α-H), 1.47–1.53 (2H, m, 11α-H, 14-H), 1.65 (1H, dm, *J* = 13.0 Hz, 11β-H), 1.73 (1H, dt, *J* = 5.1, 13.4 Hz, 1α-H), 1.82 (1H, dm, *J* = 13.0 Hz, 12β-H), 2.00 (1H, dd, *J* = 6.8, 19.1 Hz, 16α-H), 2.07 (1H, dm, *J* = 12.9 Hz, 1β-H), 2.21–2.25 (1H, m, 8-H), 2.34 (1H, dm, *J* = 18.35 Hz, 2α-H), 2.47 (1H, ddd, *J* = 1.9, 19.7 Hz, 6α-H), 2.60–2.66 (1H, m, 2β-H), 2.90–2.95 (2H, m, 6β-H, 16β-H), 4.30–4.34 (1H, m, 15β-H), 5.80 (1H, s, 4-H). ^13^C NMR (151 MHz, CD_3_OD) δ_C_: 15.8 (C-18), 17.6 (C-19), 20.8 (C-11), 32.7 (C-12), 34.4 (C-2), 35.2 (C-6), 36.2 (C-1), 38.3 (C-8), 44.5 (C-10), 46.4 (C-16), 52.8 (C-13), 59.2 (C-14), 59.6 (C-9), 70.4 (C-15), 122.9 (C-4), 182.5 (C-5), 202.1 (C-3), 218.4 (C-17). EI-MS m/z 288 [M]^+^ (24), 260 (25), 246 (100), 199 (12), 185 (11), 159 (16), 133 (17), 105 (23), 91 (47). See Figure S27 in the Supplementary Materials for the MS spectrum. Retention time: *R_t_* = 5.76 min.

**6α-Hydroxy-B-norandrost-4-en-3,17-dione (6)**: (25 mg, 29% mol.); mp 212 °C (from acetone) (lit. [40] 205–210 °C). ^1^H NMR (600 MHz, CD_3_OD) δ_H_: 0.96 (3H, s, 18-Me), 1.14 (1H, s, 19-Me), 1.23–1.28 (1H, m, 12α-H), 1.40–1.45 (1H, m, 9-H), 1.51 (1H, ddd, *J* = 3.9, 12.7, 25.4 Hz, 11α-H), 1.63–1.66 (1H, m, 11β-H), 1.76–1.83 (4H, m, 1α-H, 12β-H, 14-H, 15β-H), 2.06–2.14 (3H, m, 1β-H, 8-H, 16α-H), 2.19–2.23 (1H, m, 15α-H), 2.36 (1H, dm, *J* = 17.8 Hz, 2α-H), 2.47 (1H, dd, *J* = 0.6, 19.0 Hz, 16β-H), 2.62–2.68 (1H, m, 2β-H), 4.68 (1H, dd, *J* = 1.0; 7.0 Hz, 6β-H), 6.03 (1H, s, 4-H). ^13^C NMR (151 MHz, CD_3_OD) δ_C_: 14.5 (C-18), 17.7 (C-19), 21.0 (C-11), 22.8 (C-15), 32.3 (C-12), 34.6 (C-2), 36.5 (C-16), 36.9 (C-1), 44.2 (C-10), 44.6 (C-8), 47.0 (C-14), 50.2 (C-13), 55.4 (C-9), 71.9 (C-6), 124.6 (C-4), 183.0 (C-5), 202.9 (C-3), 222.8 (C-17). EI-MS m/z 288 [M]^+^ (98), 260 (95), 243 (70), 243 (70), 231 (66), 213 (30), 133 (50), 110 (61), 91 (79). See Figure S28 in the Supplementary Materials for the MS spectrum. Retention time: *R_t_* = 4.99 min.

### 3.8. Time Course Experiments

The conditions of time course experiments were identical to those in the main biotransformation method. At regular intervals, 5 mL samples of the reaction mixture were taken, extracted with chloroform and analyzed as described in the general experimental procedure.

### 3.9. Pharmacokinetics and Biological Activity Prediction

The assessment of the pharmacokinetic and physicochemical properties, medical usefulness and potential biological activity of the transformation substrates and the obtained derivatives was based on calculations using the SwissADME (http://www.swissadme.ch/index.php (accessed on 22 February 2024)) and Way2Drug PASS Online (http://www.way2drug.com/PASSOnline/predict.php, (accessed on 1 March 2024)) platforms. The structural formulas of the molecules were constructed using the Marvin JS ChemAxon program and saved in the SMILES format. These files were then imported into the services for analysis. In PASS Online, biological activity types are presented as the probability to be revealed (P_a_) and not to be revealed (P_i_), with independent values within the range from 0 to 1.

## 4. Conclusions

The conducted research demonstrated the suitability of the *F. culmorum* AM 282 strain for the hydroxylation of B-nor analogues of androstenedione and dehydroepiandrosterone. The reactions occurred with regio- and stereoselectivity typical of this strain in relation to the corresponding natural hormones of the standard 6:6 A/B series. With high yield, 15α-hydroxy-B-nor-DHEA was obtained as the sole reaction product after transformation of B-nor-DHEA and its acetate. The regioselectivity of hydroxylation was lower in the B-nor-AD transformation, in which, in addition to the 15α-hydroxy, a 6α-hydroxy derivative was also obtained. A detailed analysis of spatial models of DHEA, AD and their B-nor analogues, supported by time course analysis of the transformations, suggests that 7α-, 15α- and 6α-hydroxylation may be the result of the activity of the same enzyme.

Based on the model of spatial orientation of hydrogen atoms in the above-mentioned substrates and assuming that the heme oxygen of the hydroxylase is located between C-7 and C-15 atoms of the steroid molecule, the atom 15α-H in B-nor-DHEA (**1**) should be closer to this oxygen than 7α-H in DHEA and 15α-H in AD. It allows for the explanation of the preferential hydroxylation of **1** at this position. At the same time, it seems that apart from the undoubted similarities (15α-hydroxylation), the different deflection of the 7α-H bond in AD and the 6α-H bond in B-nor-AD (**3**) towards the hypothetical active center of the hydroxylase is the reason for the different regioselectivity of hydroxylation in the B ring of both molecules. As in the case of all 4-ene and 5-ene steroidal olefins, the hydroxylation of the allyl position was favored in the transformation of B-nor-AD (**3**). The results and considerations presented above may constitute a basis for the research on understanding the molecular nature of cytochrome P-450 monooxygenase present in this microorganism. A detailed analysis of the selectivity of steroid hydroxylation by *F. culmorum* at the molecular level will be possible when we have determined the genome of this strain. This will enable us to identify and isolate the enzyme and perform molecular docking experiments in the next steps. Such research will contribute to the development of a highly selective biocatalyst for steroidal 15α-hydroxylation. This type of study was recently published for cytochrome P-450 with steroidal 12β-hydroxylation activity from *Fusarium graminearum* [55].

The obtained metabolites are compounds with pharmacological potential, predicted using cheminformatics tools (Swiss-ADME and PASS online biological prediction analysis). These activities should be confirmed in the future through in vitro testing in cellular or tissue studies, and after further validation in in vivo studies to assess the broad interactions of the investigated molecules between organs and their effects on the organism as a whole. On the other hand, the introduction of a hydroxyl group into the steroid structure creates the possibility of subsequent structural modifications of the molecules in the search for new substances with promising applications in both biotechnology and pharmacy.

## Figures and Tables

**Table 1:** Probability of metabolization of compounds **1**–**6** by cytochromes P450 predicted using PASS Online tool.

Compound	Pass Prediction for CYP Substrate							
		2B5	2C12	2J2	2A1	3A1	2A4	2A11
1	P_a_	0.887	0.960	0.951	0.883	0.877	0.909	0.846
	P_i_	0.002	0.003	0.002	0.002	0.004	0.001	0.003
2	P_a_	0.901	0.913	0.922	0.818	0.901	0.854	0.807
	P_i_	0.002	0.009	0.003	0.003	0.003	0.002	0.003
3	P_a_	0.977	0.967	0.958	0.950	0.930	0.927	0.910
	P_i_	0.000	0.003	0.002	0.001	0.002	0.001	0.002
4	P_a_	0.817	0.932	0.920	0.771	0.875	0.855	0.788
	P_i_	0.004	0.006	0.003	0.003	0.004	0.002	0.004
5	P_a_	0.929	0.967	0.960	0.916	0.945	0.926	0.873
	P_i_	0.002	0.003	0.001	0.002	0.002	0.001	0.002
6	P_a_	0.920	0.964	0.955	0.902	0.897	0.917	0.845
	P_i_	0.002	0.003	0.002	0.002	0.003	0.001	0.003

P_a_—probable activity; P_i_—probable inactivity. Value 1 represents a 100% probability of P_a_ or P_i._

**Table 2:** Probability of interactions of compounds **1**–**6** with selected enzymes predicted using PASS Online tool.

Activity		Number of Compound					
		1	2	4	3	5	6
Testosterone 17β-dehydrogenase (NADP^+^) inhibitor	P_a_	0.977	0.949	0.963	0.981	0.982	0.979
	P_i_	0.001	0.003	0.002	0.001	0.001	0.001
27-Hydroxycholesterol 7α-monooxygenase inhibitor	P_a_	0.973	0.936	0.955	0.956	0.943	0.936
	P_i_	0.001	0.002	0.001	0.001	0.002	0.002
Prostaglandin-E2 9-reductase inhibitor	P_a_	0.910	0.876	0.933	0.907	0.959	0.896
	P_i_	0.004	0.006	0.003	0.004	0.002	0.005
Lysase inhibitor	P_a_	0.873	0.808	0.788	0.922	0.897	0.911
	P_i_	0.004	0.009	0.011	0.003	0.004	0.003
Morphine 6-dehydrogenase inhibitor	P_a_	0.847	0.626	0.776	0.809	0.893	0.805
	P_i_	0.001	0.003	0.001	0.001	0.001	0.001
Sulfotransferase substrate	P_a_	0.912	0.857	0.902	0.800	0.890	0.738
	P_i_	0.002	0.003	0.002	0.004	0.003	0.004
UDP-glucuronosyltransferase substrate	P_a_	0.847	0.759	0.880	0.819	0.942	0.862
	P_i_	0.004	0.010	0.003	0.005	0.003	0.004
UGT1A substrate	P_a_	0.858	0.725	0.851	0.814	0.911	0.868
	P_i_	0.003	0.008	0.004	0.004	0.003	0.003

P_a_—probable activity; P_i_—probable inactivity. Value 1 represents a 100% probability of P_a_ or P_i._

**Table 3:** Biological activity predictions for B-nor substrates **1**–**3** and their hydroxymetabolites **4**–**6** using the PASS Online tool.

Activity		Number of Compound					
		1	2	4	3	5	6
Ovulation inhibitor	P_a_	0.917	0.897	0.849	0.949	0.917	0.873
	P_i_	0.002	0.002	0.003	0.001	0.002	0.002
Respiratory analeptic	P_a_	0.818	0.858	0.872	0.844	0.938	0.706
	P_i_	0.007	0.005	0.005	0.006	0.004	0.014
Cholesterol antagonist	P_a_	0.878	0.837	0.834	0.801	0.835	0.818
	P_i_	0.003	0.004	0.004	0.004	0.004	0.004
Protein synthesis stimulant	P_a_	0.823	0.839	0.725	0.771	0.768	0.743
	P_i_	0.001	0.001	0.001	0.001	0.001	0.001
Menopausal disorder treatment	P_a_	0.832	0.773	0.771	0.747	0.784	0.700
	P_i_	0.002	0.002	0.002	0.003	0.002	0.003
Antihypercholesterolemic	P_a_	0.802	0.781	0.718	0.789	0.802	0.682
	P_i_	0.005	0.005	0.006	0.005	0.005	0.009

P_a_—probable activity; P_i_—probable inactivity. Value 1 represents a 100% probability of P_a_ or P_i._

## Data Availability

Samples of compounds **4**–**6** are available from the authors.

## Supplementary Materials

The following supporting information can be downloaded at: https://www.mdpi.com/article/10.3390/ijms252211913/s1.
