# Supplementary material for: Modification of B-Nor Steroids Mediated by Filamentous Fungus Fusarium culmorum: Focus on 15α-Hydroxylase Activity
Source: Int J Mol Sci. 2024 Nov 6;25(22):11913. doi: 10.3390/ijms252211913 (PMC11594044; doi:10.3390/ijms252211913)
Supplement: Supplementary file 1 [file ijms-25-11913-s001.zip › ijms-3251165-supplementary.pdf]

Supplementary data

Modification of B-nor steroids mediated by filamentous fungus *Fusarium culmorum*: focus on 15α-hydroxylase activity

Alina Świzdor*, Tomasz Janeczko and Anna Panek*

Department of Food Chemistry and Biocatalysis, Wrocław University of Environmental and Life Sciences, C.K. Norwida 25, Wrocław 50-375, Poland; alina.swizdor@upwr.edu.pl (A.Ś.); anna.panek@upwr.edu.pl (A.P.); tomasz.janeczko@upwr.edu.pl (T.J.)

Contens:

Figure S1. ^{1}H NMR spectrum of 3β-hydroxy-B-norandrost-5-en-17-one (**1**)

Figure S2. ^{13}C NMR spectrum of 3β-hydroxy-B-norandrost-5-en-17-one (**1**)

Figure S3. DEPT NMR spectrum of 3β-hydroxy-B-norandrost-5-en-17-one (**1**)

Figure S4. HSQC NMR spectrum of 3β-hydroxy-B-norandrost-5-en-17-one (**1**)

Figure S5. ^{1}H NMR spectrum of B-norandrost-4-en-3,17-dione (**3**)

Figure S6. ^{13}C NMR spectrum of B-norandrost-4-en-3,17-dione (**3**)

Figure S7. DEPT NMR spectrum of B-norandrost-4-en-3,17-dione (**3**)

Figure S8. HSQC NMR spectrum of B-norandrost-4-en-3,17-dione (**3**)

Figure S9. ^{1}H NMR spectrum of 3β,15α-dihydroxy-B-norandrost-5-en-17-one (**4**)

Figure S10. ^{13}C NMR spectrum of 3β,15α-dihydroxy-B-norandrost-5-en-17-one (**4**)

Figure S11. DEPT NMR spectrum of 3β,15α-dihydroxy-B-norandrost-5-en-17-one (**4**)

Figure S12. HSQC NMR spectrum of 3β,15α-dihydroxy-B-norandrost-5-en-17-one (**4**)

Figure S13. ^{1}H NMR spectrum of 15α-hydroxy-B-norandrost-4-en-3,17-dione (**5**)

Figure S14. ^{13}C NMR spectrum of 15α-hydroxy-B-norandrost-4-en-3,17-dione (**5**)

Figure S15. DEPT NMR spectrum of 15α-hydroxy-B-norandrost-4-en-3,17-dione (**5**)

Figure S16. HSQC NMR spectrum of 15α-hydroxy-B-norandrost-4-en-3,17-dione (**5**)

Figure S17. ^{1}H NMR spectrum of 6α-hydroxy-B-norandrost-4-en-3,17-dione (**6**)

Figure S18. ^{13}C NMR spectrum of 6α-hydroxy-B-norandrost-4-en-3,17-dione (**6**)

Figure S19. DEPT NMR spectrum of 6α-hydroxy-B-norandrost-4-en-3,17-dione (**6**)

Figure S20. HSQC NMR spectrum of 6α-hydroxy-B-norandrost-4-en-3,17-dione (**6**)

Figure S21. NOESY NMR spectrum of 6α-hydroxy-B-norandrost-4-en-3,17-dione (**6**)

Figure S22. *In silico* physicochemical properties (ADME profile) of hydroxy metabolites **4-6**

Figure S23. Graphic distribution of substrates **1**-**3** and their metabolites **4-6** using BOILED-Egg predictive model

Figure S24. MS spectrum of 3β-hydroxy-B-norandrost-5-en-17-one (**1**).

Figure S25. MS spectrum of B-norandrost-4-en-3,17-dione (**3**).

Figure S26. MS spectrum of 3β,15α-dihydroxy-B-norandrost-5-en-17-one (**4**).

Figure S27. MS spectrum of 15α-hydroxy-B-norandrost-4-en-3,17-dione (**5**).

Figure S28. MS spectrum of 6α-hydroxy-B-norandrost-4-en-3,17-dione (**6**).

Table S1. Composition of crude mixtures obtained in transformations of 3β-acetoxy-B-norandrost-5-en-17-one (**2**) by *F. culmorum* AM282.

Table S2. Comparison of the composition of the product mixtures obtained in transformations of B-nor-DHEA (**1**), DHEA and an equimolar mixture of both compounds by *F. culmorum* AM282.

Scheme S1. Flowchart depicting the main stages of the studies.

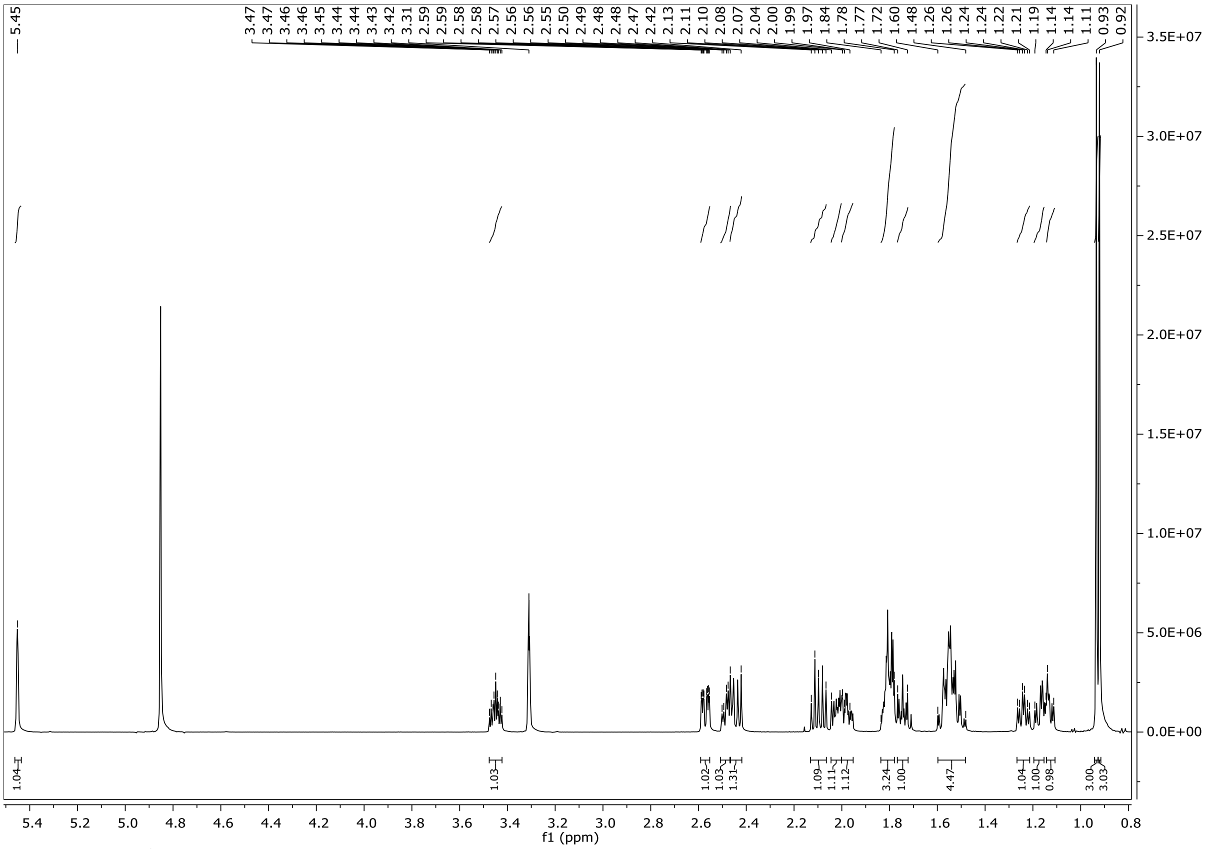

Figure S1. ^{1}H NMR spectrum of 3β-hydroxy-B-norandrost-5-en-17-one (**1**).

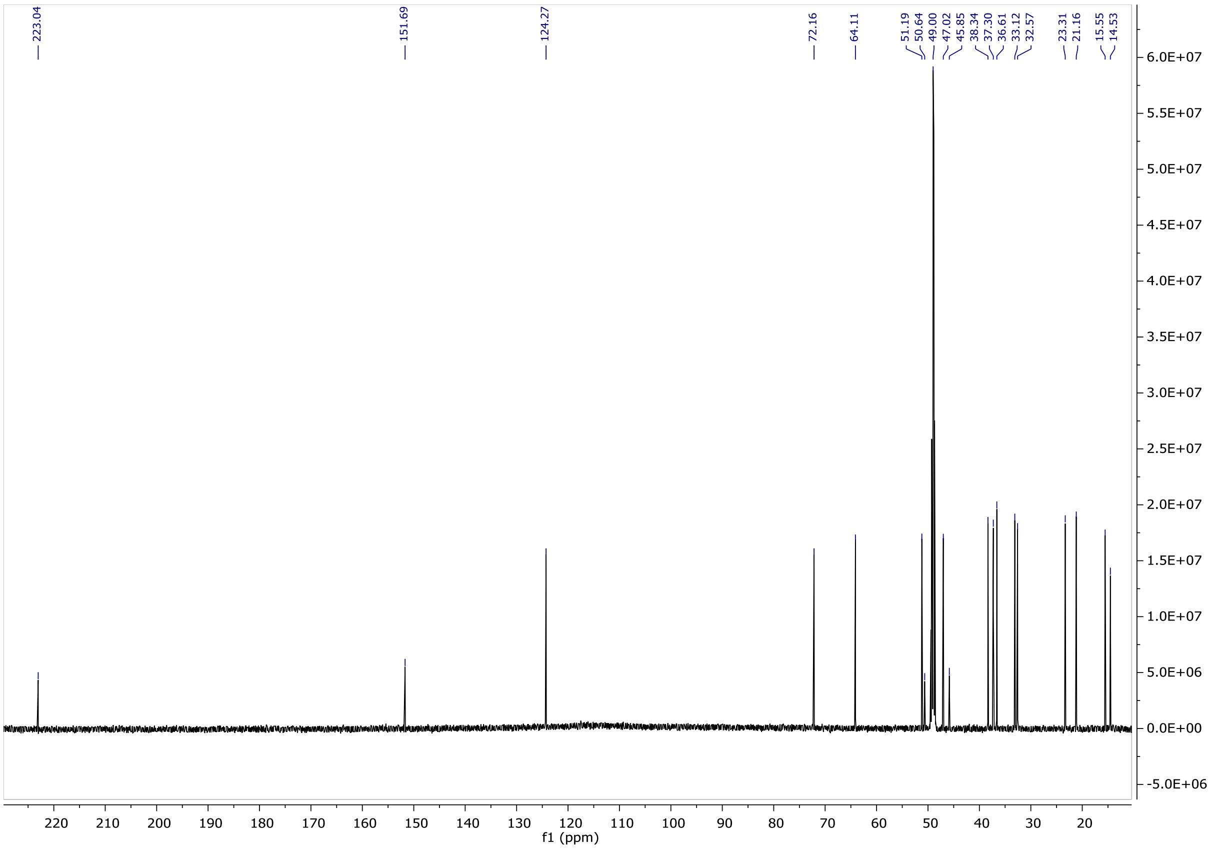

Figure S2. ^{13}C NMR spectrum of 3β-hydroxy-B-norandrost-5-en-17-one (**1**).

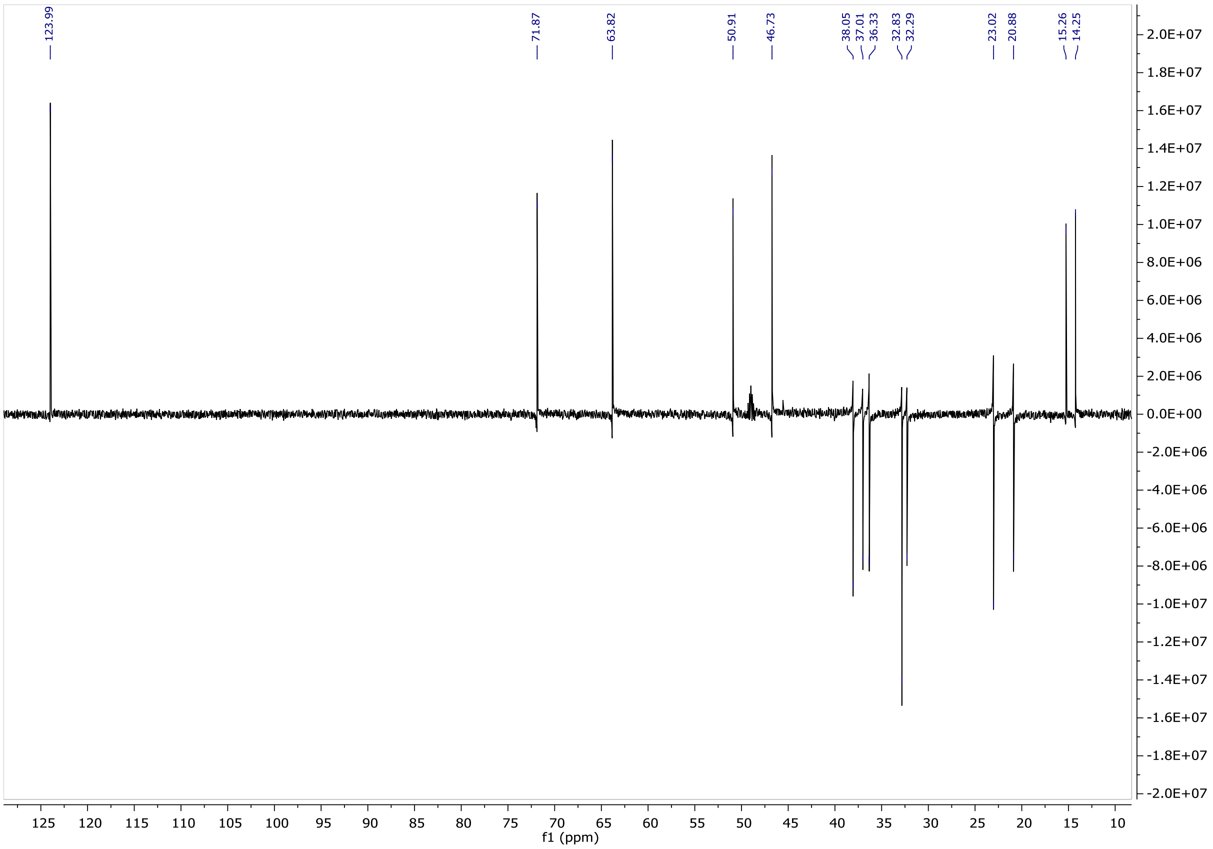

Figure S3. DEPT NMR spectrum of 3β-hydroxy-B-norandrost-5-en-17-one (**1**).

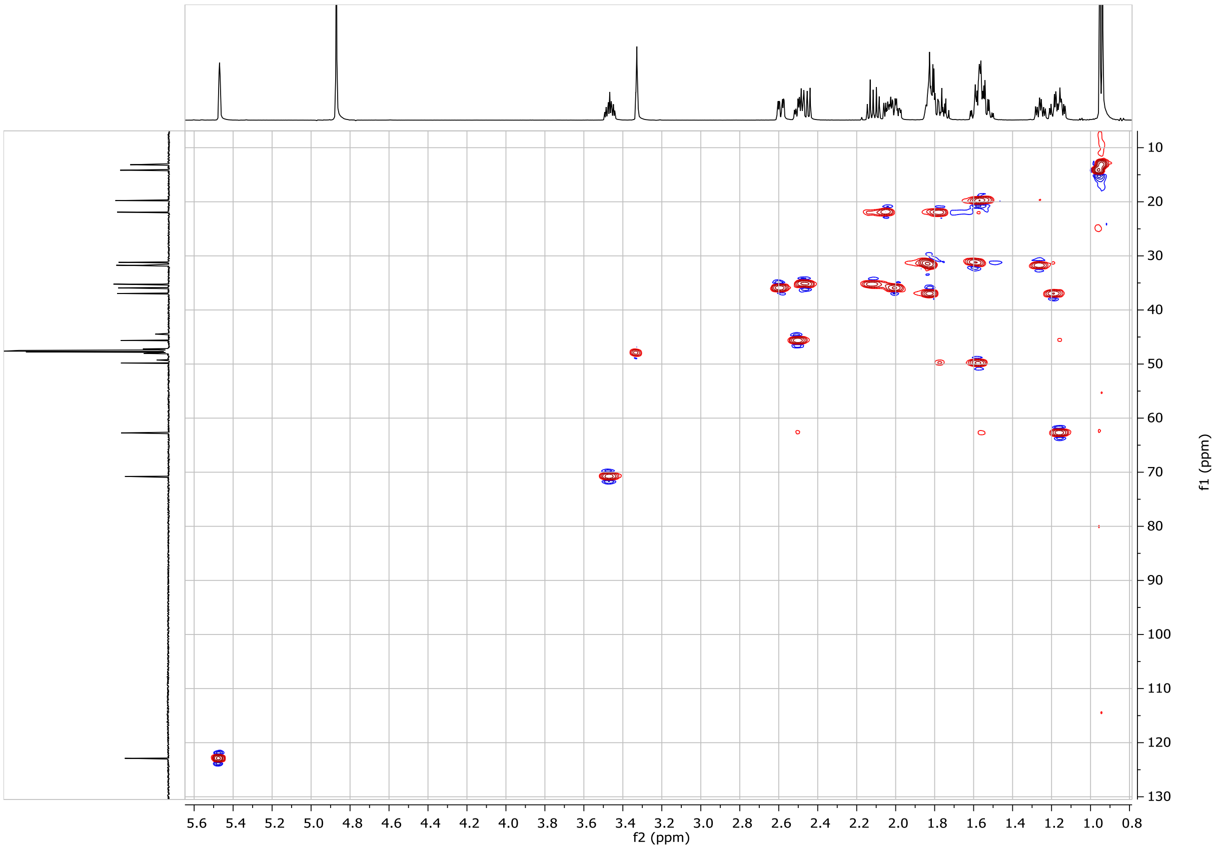

Figure S4. HSQC NMR spectrum of 3β-hydroxy-B-norandrost-5-en-17-one (**1**).

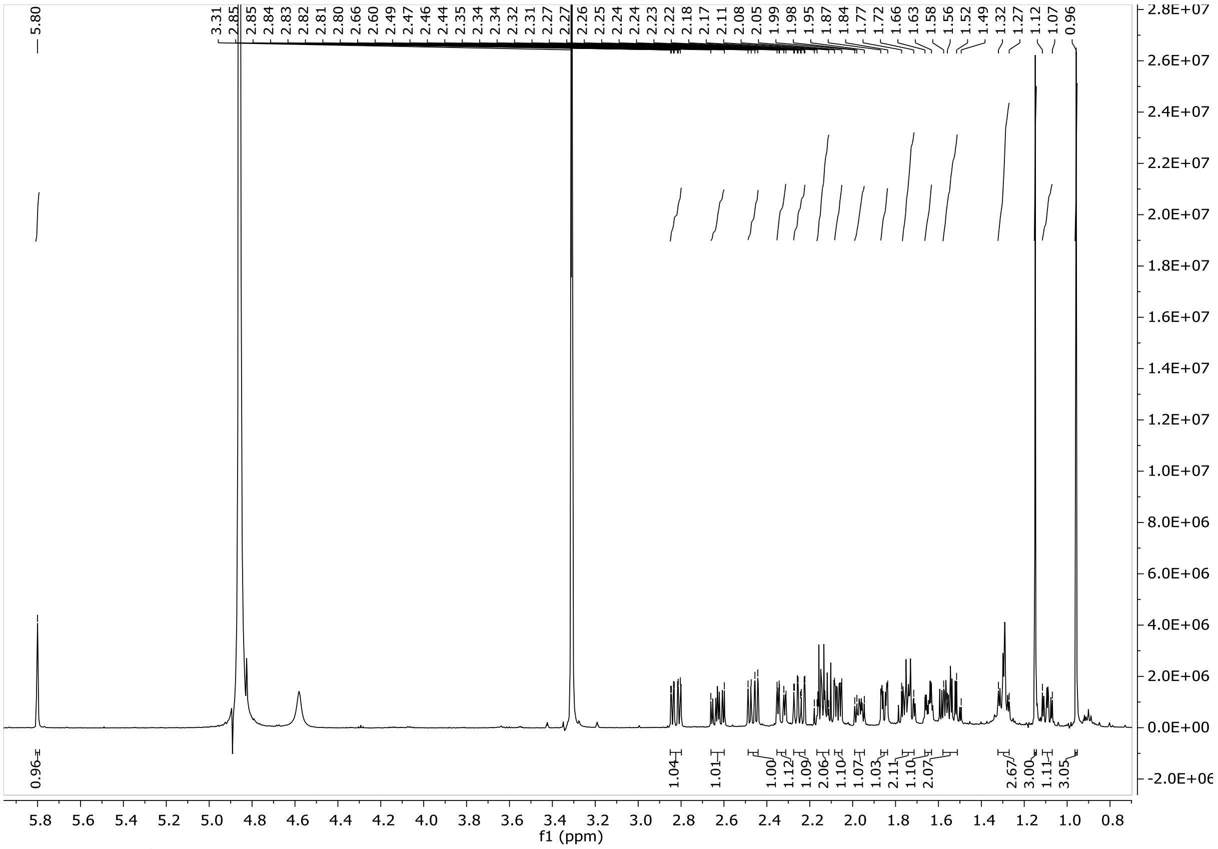

Figure S5. ^{1}H NMR spectrum of B-norandrost-4-en-3,17-dione (**3**).

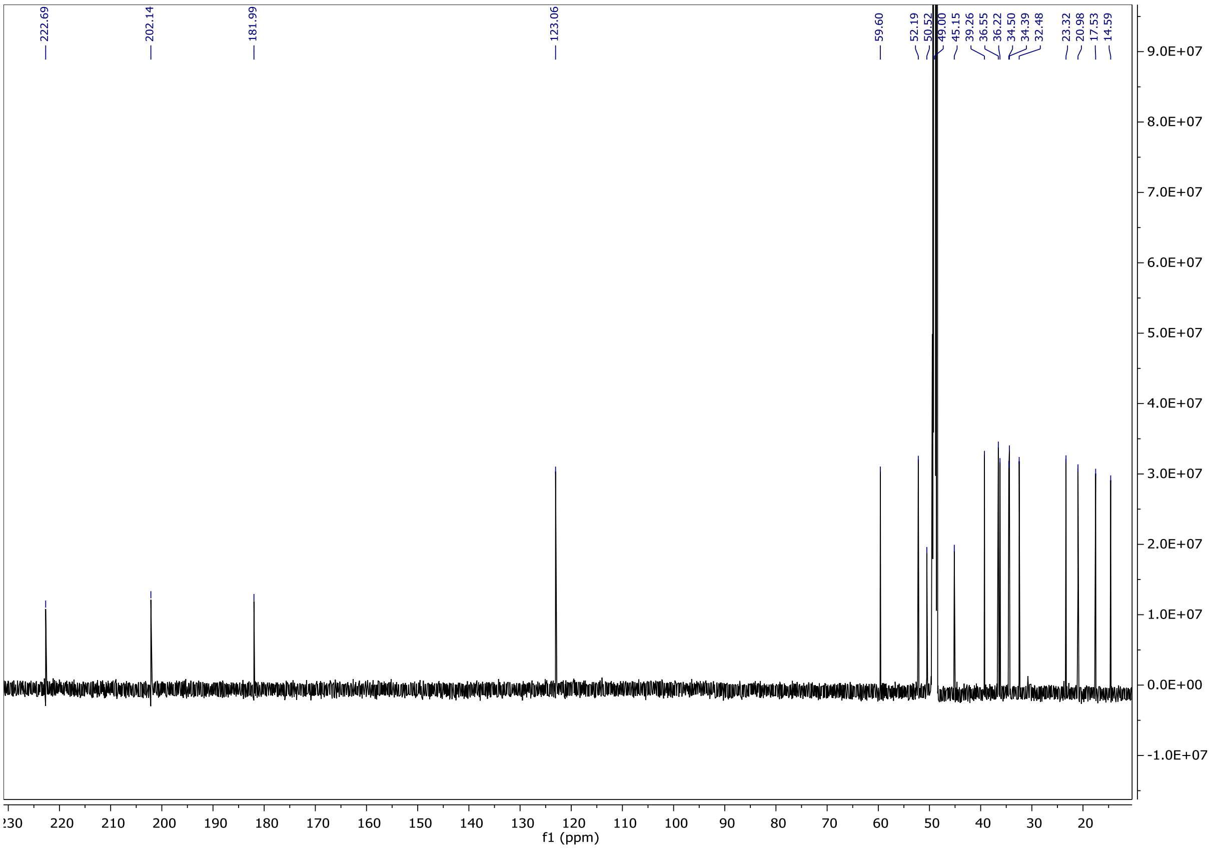

Figure S6.^{13}C NMR spectrum of B-norandrost-4-en-3,17-dione (**3**).

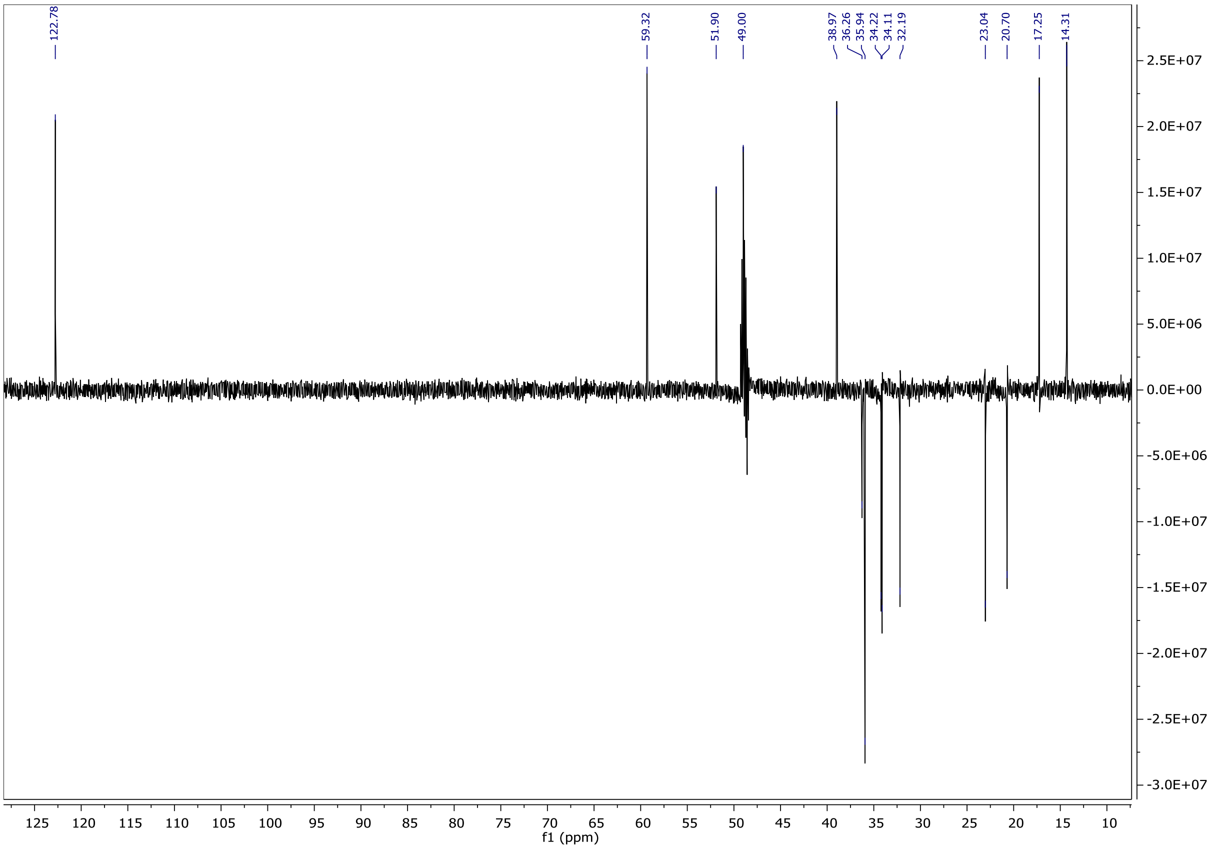

Figure S7. DEPT NMR spectrum of B-norandrost-4-en-3,17-dione (**3**).

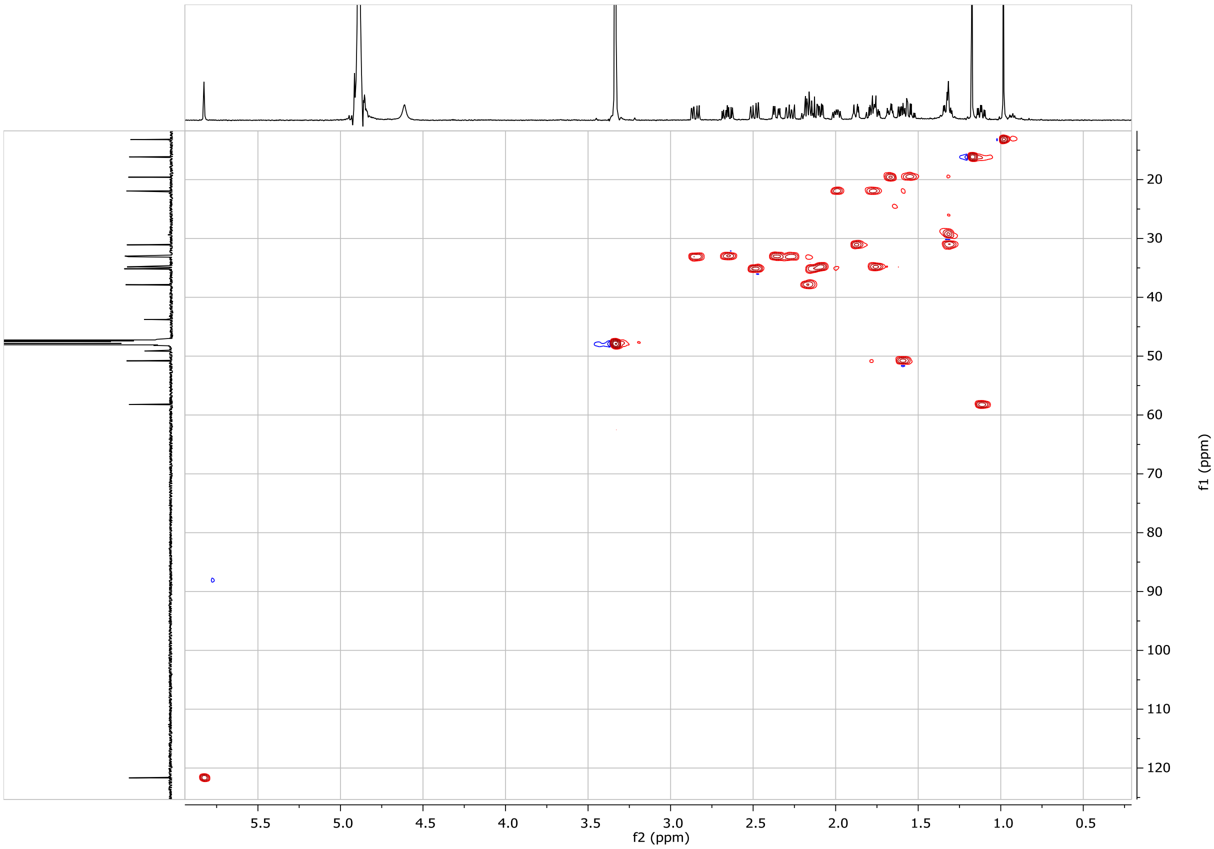

Figure S8. HSQC NMR spectrum of B-norandrost-4-en-3,17-dione (**3**).

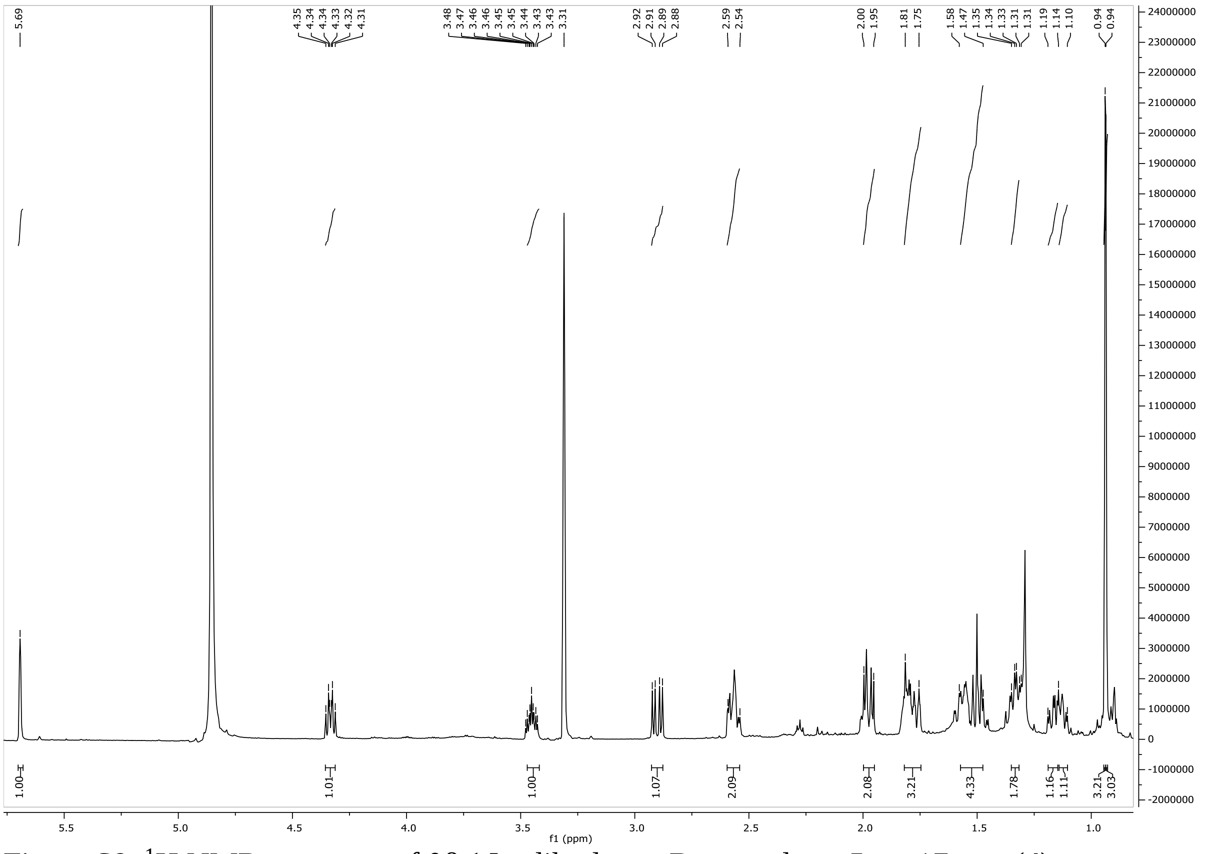

Figure S9. ^{1}H NMR spectrum of 3β,15α-dihydroxy-B-norandrost-5-en-17-one (**4**).

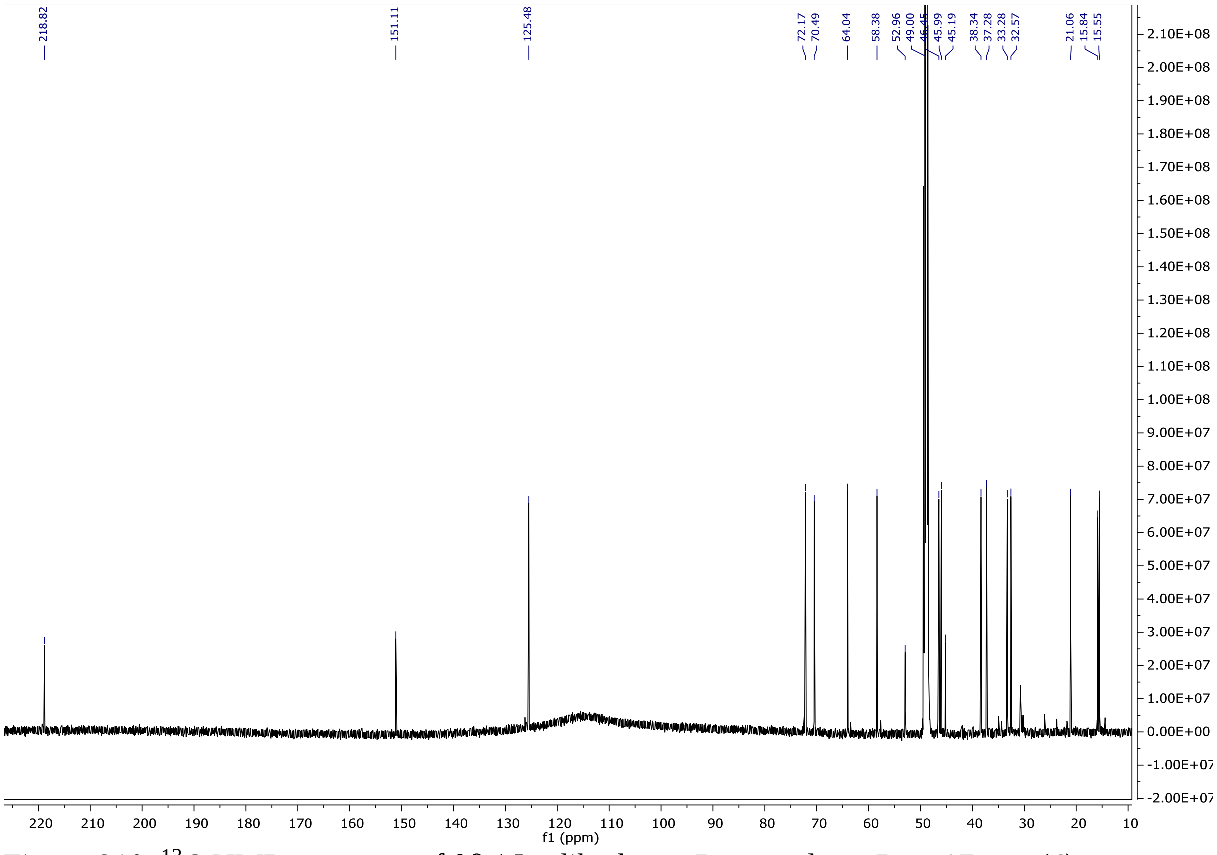

Figure S10. ^{13}C NMR spectrum of 3β,15α-dihydroxy-B-norandrost-5-en-17-one (**4**).

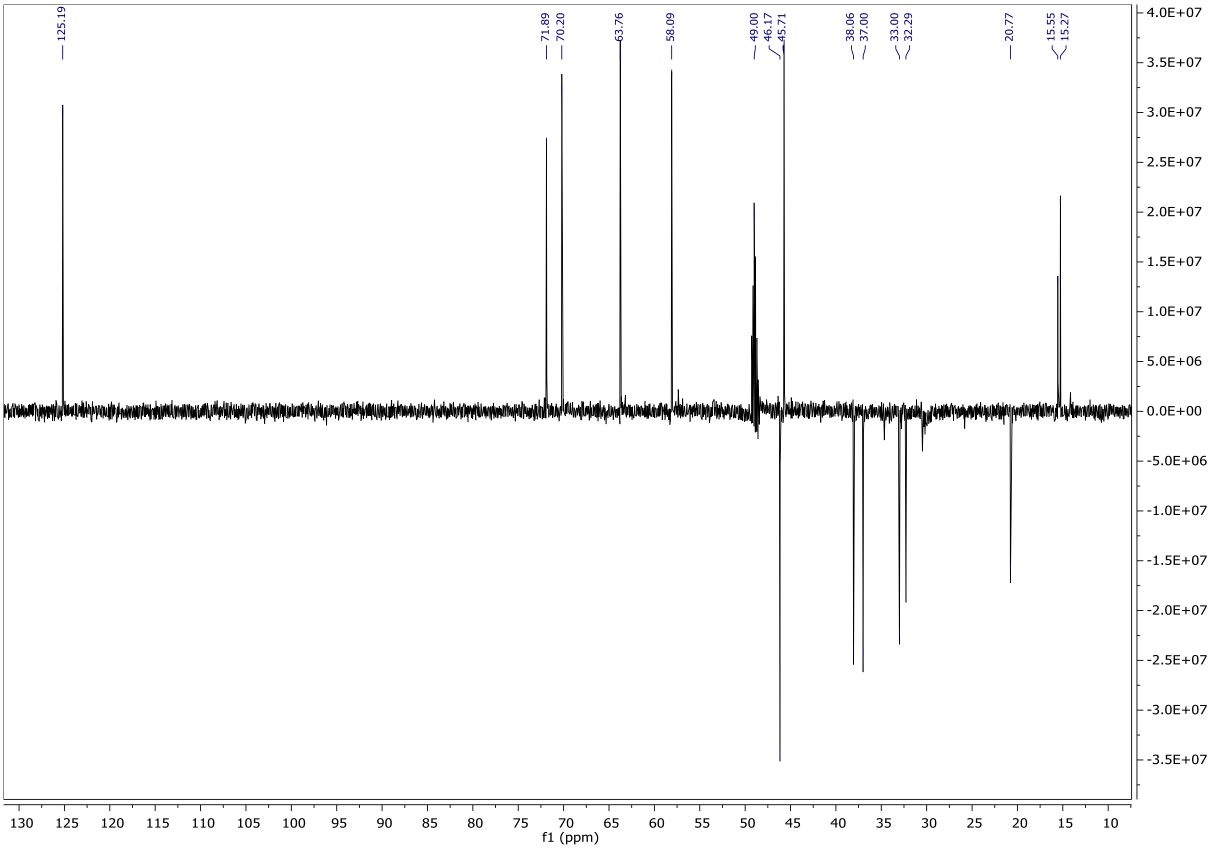

Figure S11. DEPT NMR spectrum of 3β,15α-dihydroxy-B-norandrost-5-en-17-one (**4**).

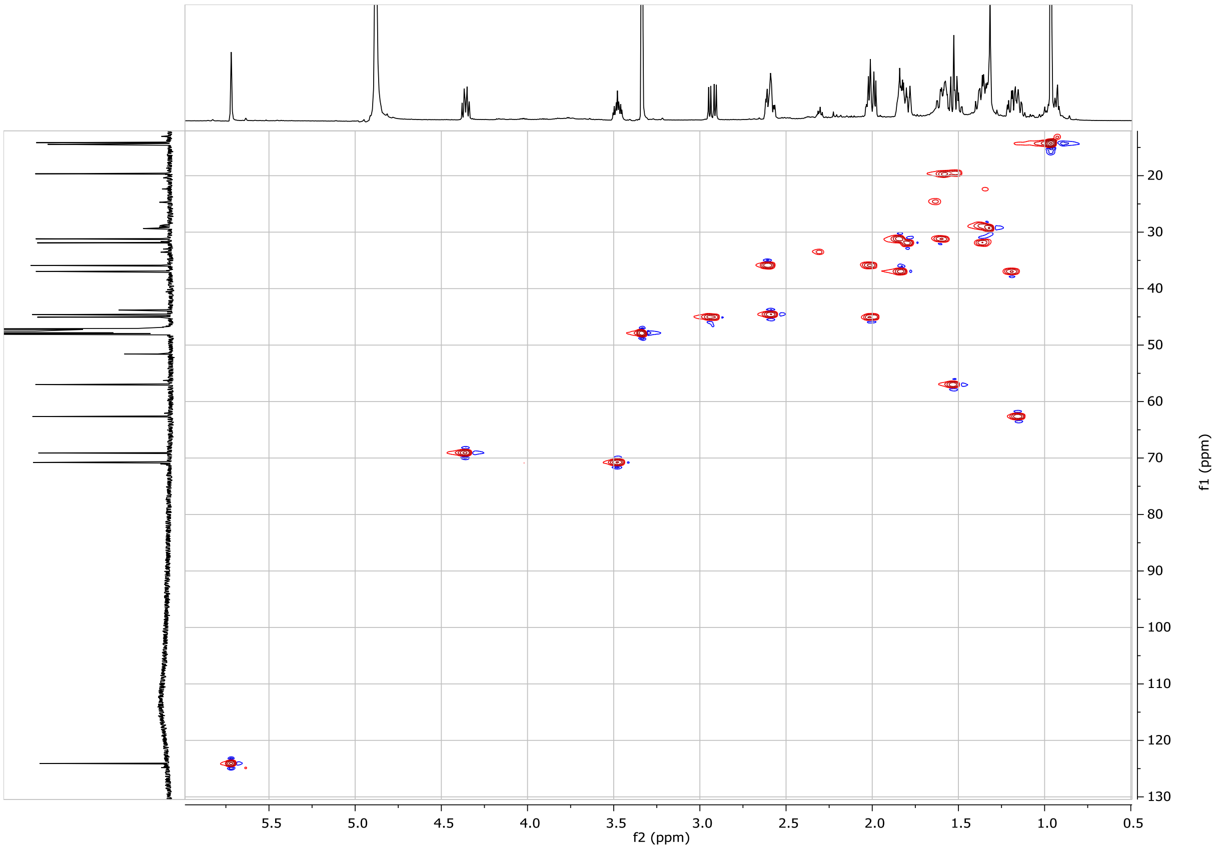

Figure S12. HSQC NMR spectrum of 3β,15α-dihydroxy-B-norandrost-5-en-17-one (**4**).

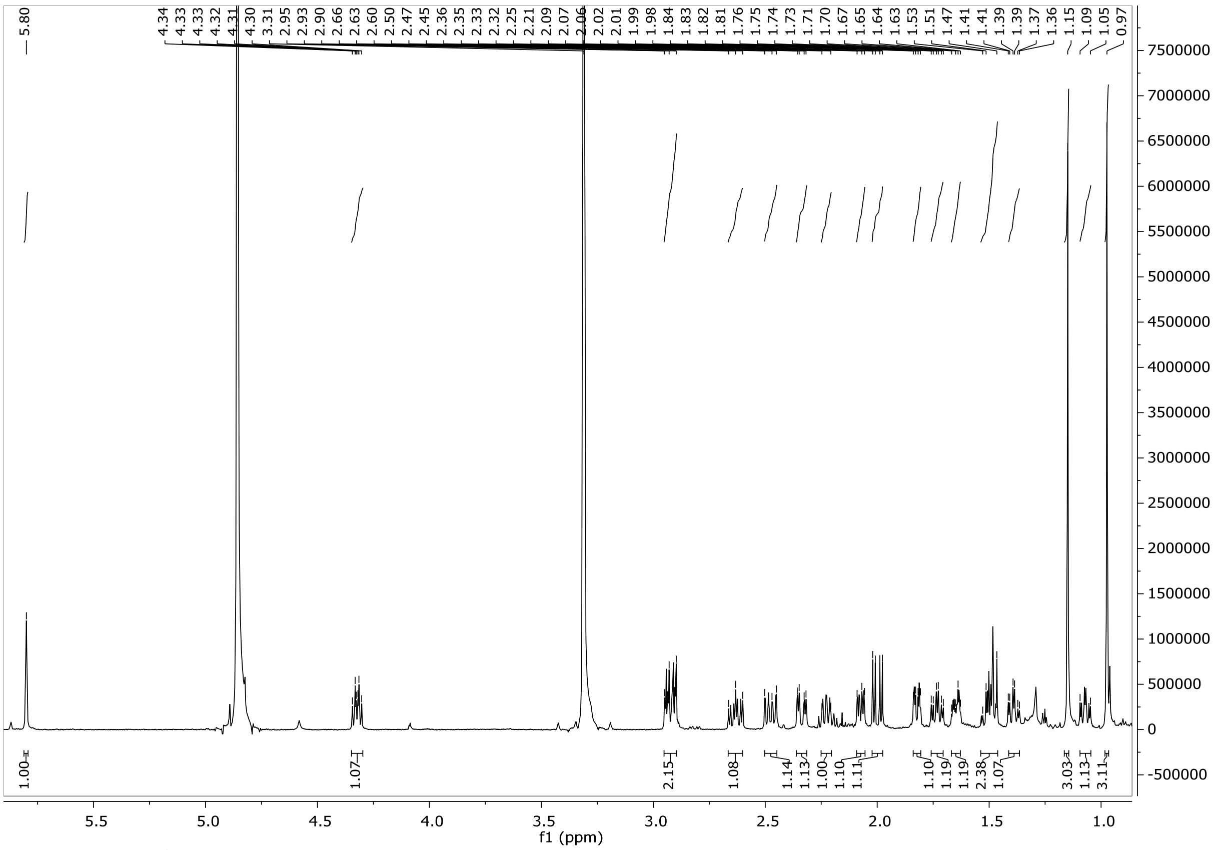

Figure S13. ^{1}H NMR spectrum of 15α-hydroxy-B-norandrost-4-en-3,17-dione (**5**).

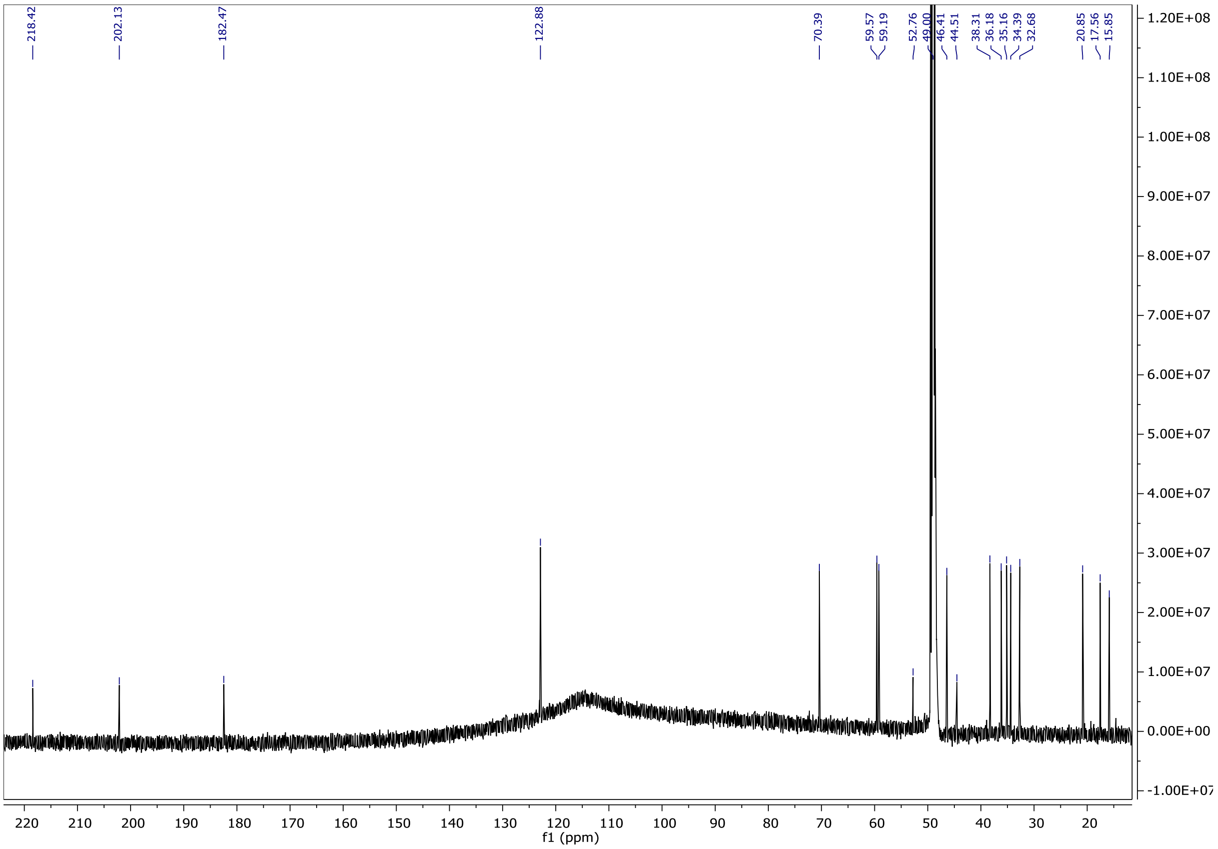

Figure S14. ^{13}C NMR spectrum of 15α-hydroxy-B-norandrost-4-en-3,17-dione (**5**).

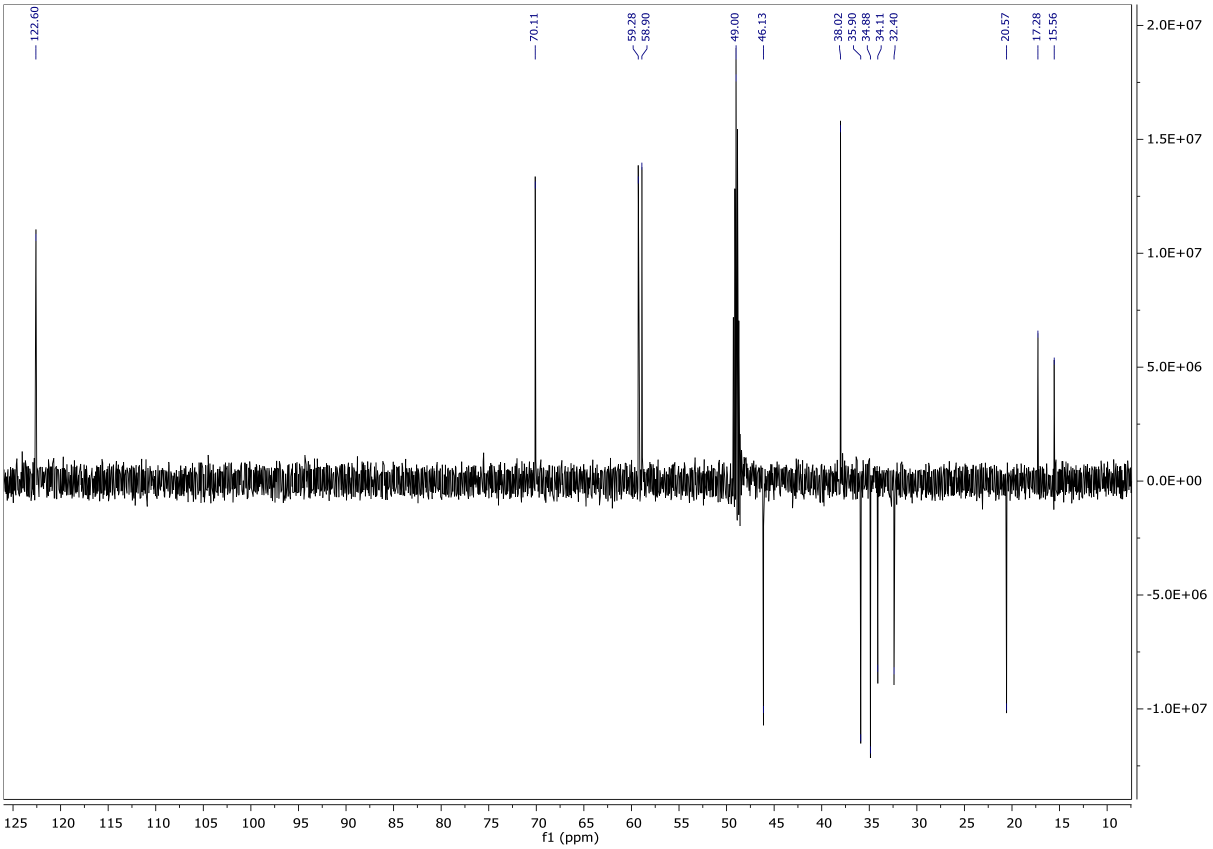

Figure S15. DEPT NMR spectrum of 15α-hydroxy-B-norandrost-4-en-3,17-dione (**5**).

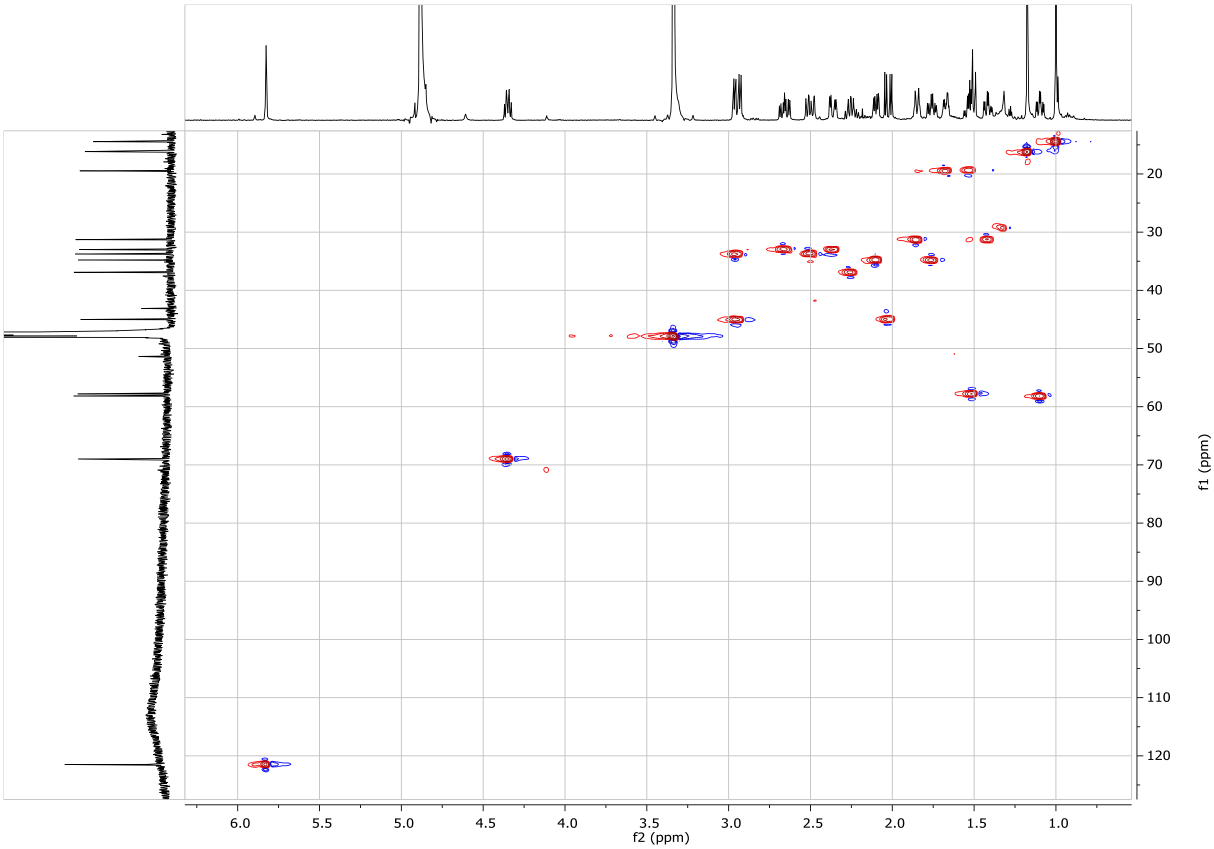

Figure S16. HSQC NMR spectrum of 15α-hydroxy-B-norandrost-4-en-3,17-dione (**5**).

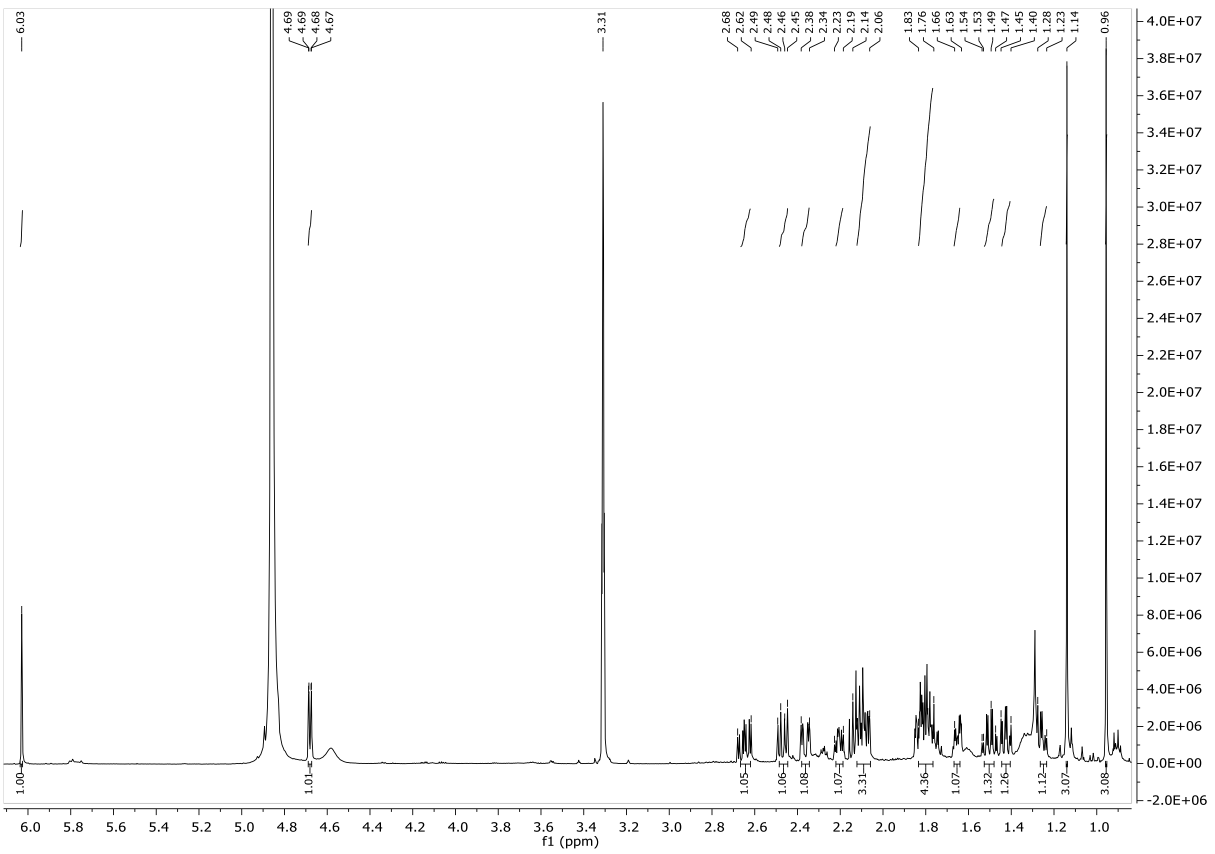

Figure S17. ^{1}H NMR spectrum of 6α-hydroxy-B-norandrost-4-en-3,17-dione (**6**).

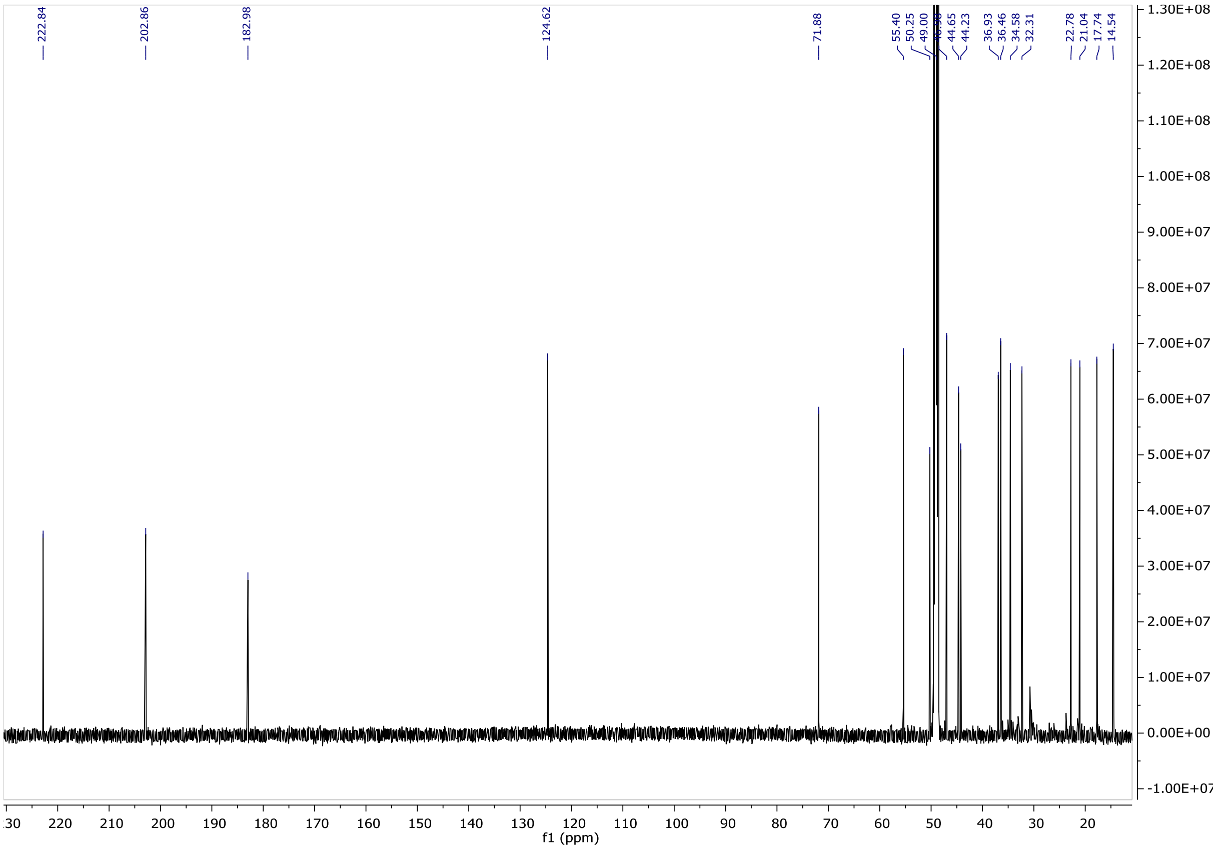

Figure S18. ^{13}C NMR spectrum of 6α-hydroxy-B-norandrost-4-en-3,17-dione (**6**).

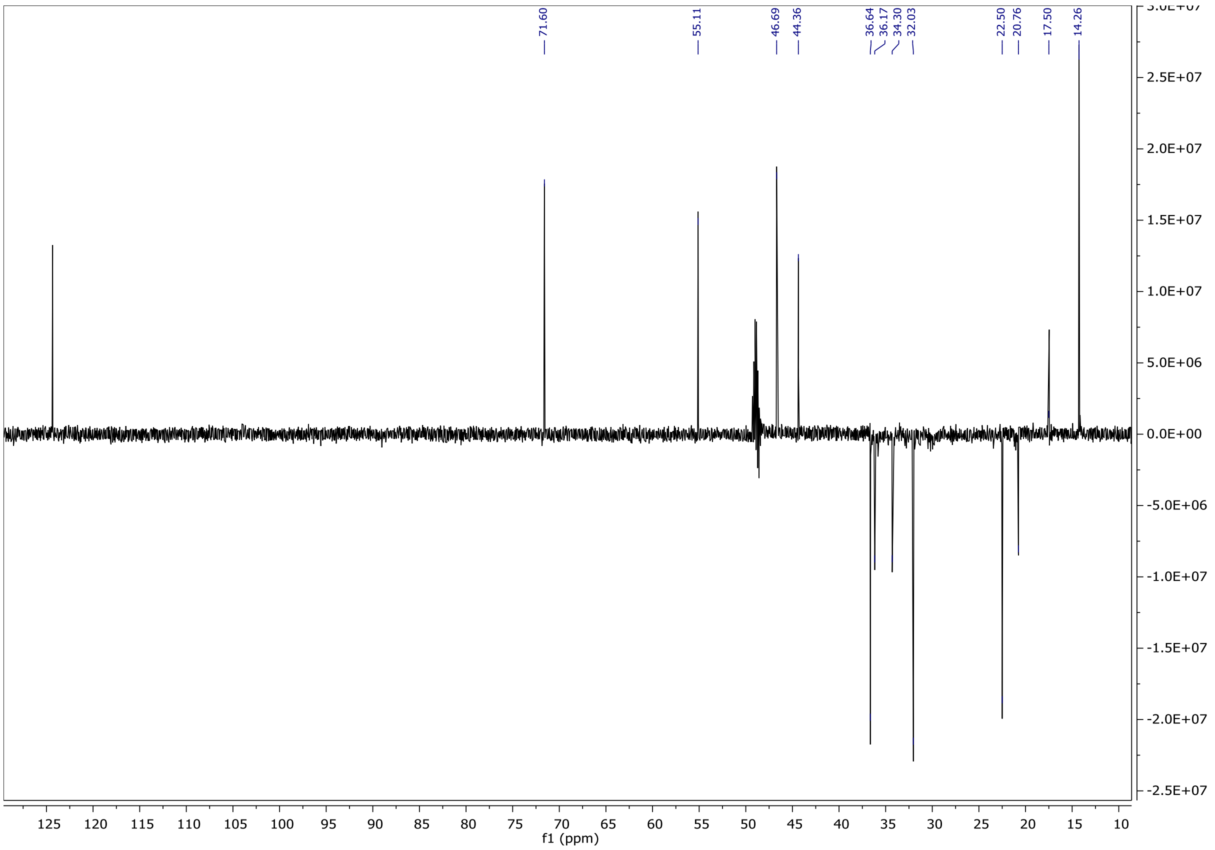

Figure S19. DEPT NMR spectrum of 6α-hydroxy-B-norandrost-4-en-3,17-dione (**6**).

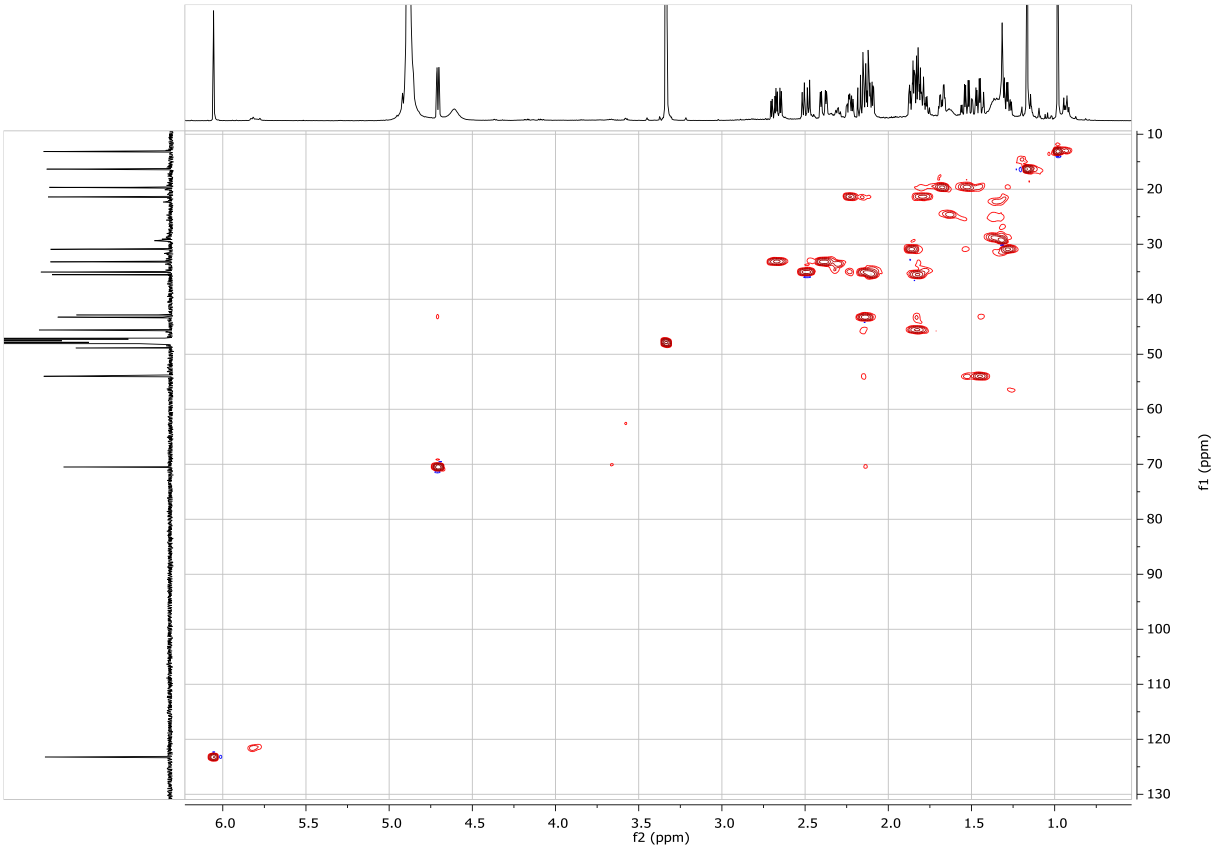

Figure S20. HSQC NMR spectrum of 6α-hydroxy-B-norandrost-4-en-3,17-dione (**6**).

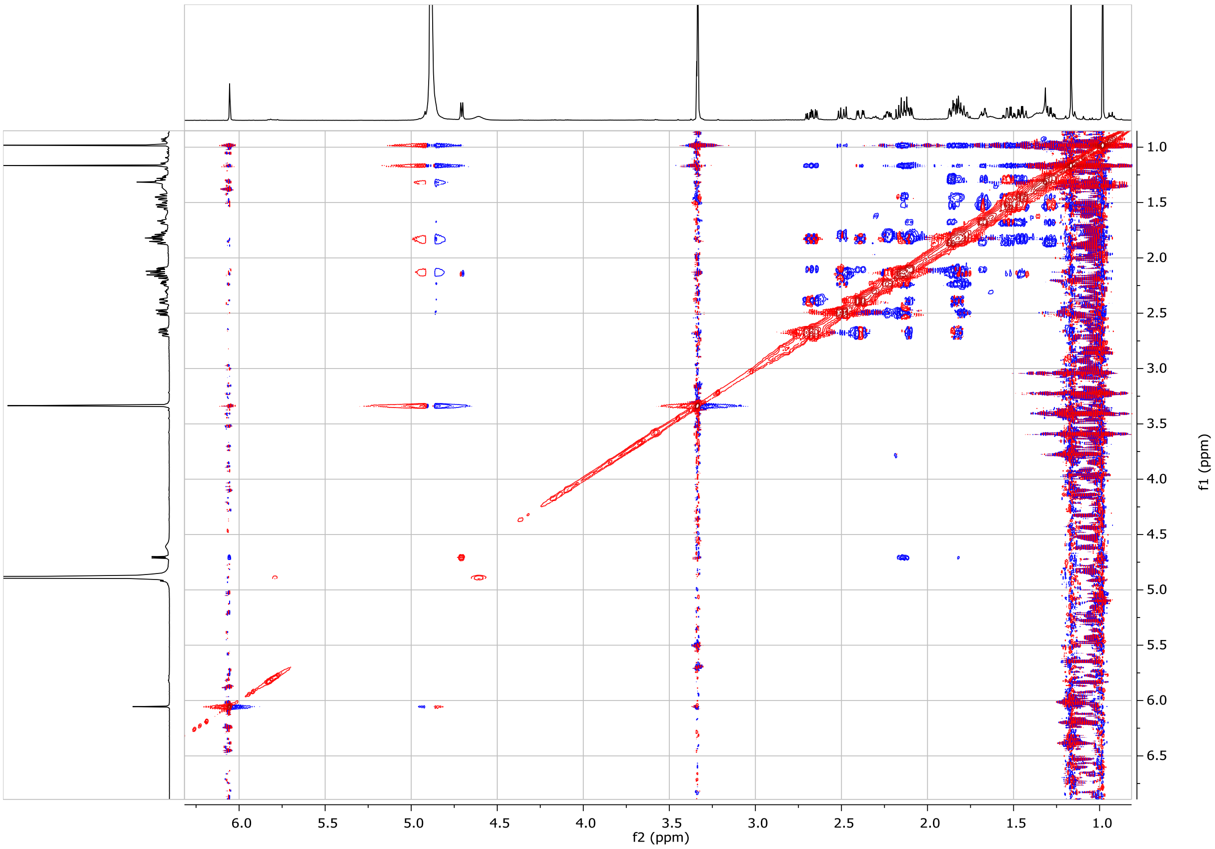

Figure S21. NOESY NMR spectrum of 6α-hydroxy-B-norandrost-4-en-3,17-dione (**6**).

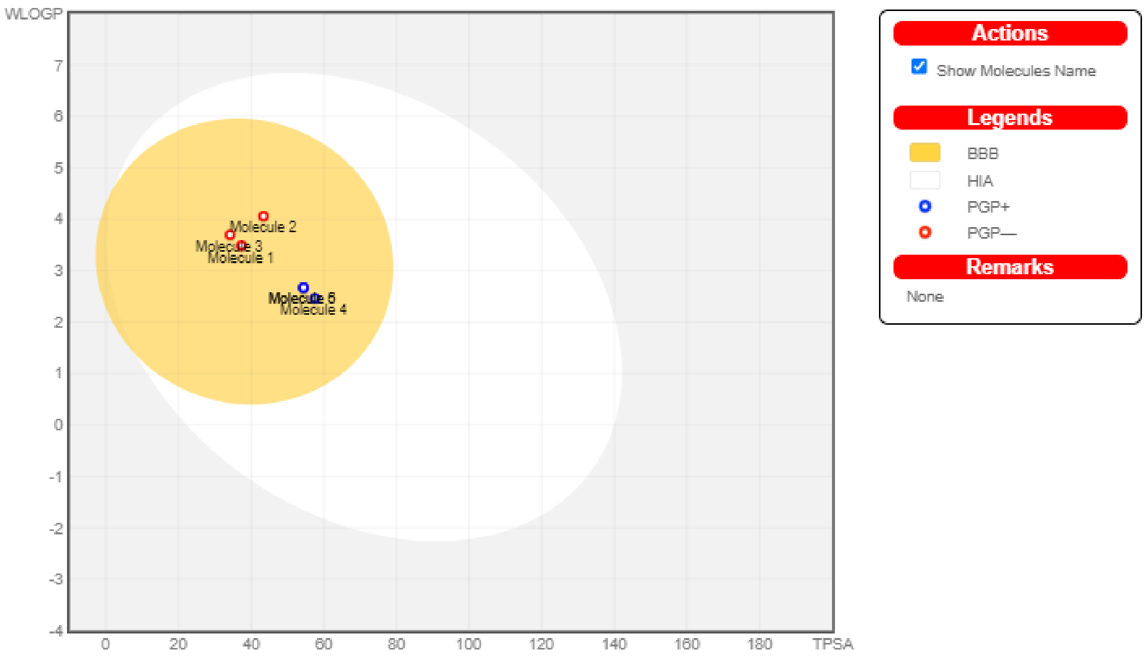

Figure S22. Graphic distribution of substrates **1-3** and their metabolites **4-6** using BOILED-Egg predictive model

Molecule 4

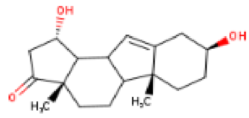

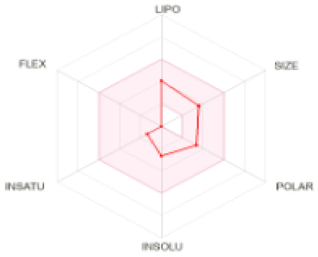

SMILES O[C@H]1CC[C@]2(C(=CC3C2CC[C@]2(C3[C@@H](O)CC2=O)C)C1)C

Physicochemical Properties	
Formula	C18H26O3
Molecular weight	290.40 g/mol
Num. heavy atoms	21
Num. arom. heavy atoms	0
Fraction Csp3	0.83
Num. rotatable bonds	0
Num. H-bond acceptors	3
Num. H-bond donors	2
Molar Refractivity	81.71
TPSA	57.53 Å²

Lipophilicity	
Log $P_{o/w}$ (iLOGP)	2.51
Log $P_{o/w}$ (XLOGP3)	1.61
Log $P_{o/w}$ (WLOGP)	2.46
Log $P_{o/w}$ (MLOGP)	2.48
Log $P_{o/w}$ (SILICOS-IT)	2.43
Consensus Log $P_{o/w}$	2.30

Water Solubility	
Log *S* (ESOL)	-2.65
Solubility	6.43e-01 mg/ml ; 2.21e-03 mol/l
Class	Soluble
Log *S* (Ali)	-2.43
Solubility	1.08e+00 mg/ml ; 3.72e-03 mol/l
Class	Soluble
Log *S* (SILICOS-IT)	-2.40
Solubility	1.16e+00 mg/ml ; 3.99e-03 mol/l
Class	Soluble

Pharmacokinetics	
GI absorption	High
BBB permeant	Yes
P-gp substrate	Yes
CYP1A2 inhibitor	No
CYP2C19 inhibitor	No
CYP2C9 inhibitor	No
CYP2D6 inhibitor	No
CYP3A4 inhibitor	No
Log K_p (skin permeation)	-6.93 cm/s

Druglikeness	
Lipinski	Yes; 0 violation
Ghose	Yes
Veber	Yes
Egan	Yes
Muegge	Yes
Bioavailability Score	0.55

Medicinal Chemistry	
PAINS	0 alert
Brenk	1 alert: isolated_alkene
Leadlikeness	Yes
Synthetic accessibility	4.99

Molecule 5

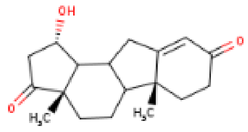

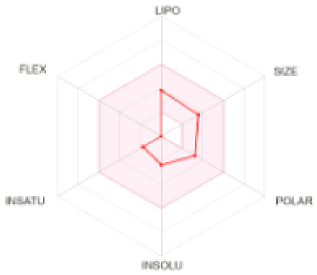

SMILES O=C1CC[C@]2(C(=C1)CC1C2CC[C@]2(C1[C@@H](O)CC2=O)C)C

Physicochemical Properties	
Formula	C18H24O3
Molecular weight	288.38 g/mol
Num. heavy atoms	21
Num. arom. heavy atoms	0
Fraction Csp3	0.78
Num. rotatable bonds	0
Num. H-bond acceptors	3
Num. H-bond donors	1
Molar Refractivity	80.75
TPSA	54.37 Å²

Lipophilicity	
Log $P_{o/w}$ (iLOGP)	2.36
Log $P_{o/w}$ (XLOGP3)	1.23
Log $P_{o/w}$ (WLOGP)	2.67
Log $P_{o/w}$ (MLOGP)	2.39
Log $P_{o/w}$ (SILICOS-IT)	3.01
Consensus Log $P_{o/w}$	2.33

Water Solubility	
Log *S* (ESOL)	-2.40
Solubility	1.14e+00 mg/ml ; 3.95e-03 mol/l
Class	Soluble
Log *S* (Ali)	-1.97
Solubility	3.09e+00 mg/ml ; 1.07e-02 mol/l
Class	Very soluble
Log *S* (SILICOS-IT)	-3.09
Solubility	2.32e-01 mg/ml ; 8.05e-04 mol/l
Class	Soluble

Pharmacokinetics	
GI absorption	High
BBB permeant	Yes
P-gp substrate	Yes
CYP1A2 inhibitor	No
CYP2C19 inhibitor	No
CYP2C9 inhibitor	No
CYP2D6 inhibitor	No
CYP3A4 inhibitor	No
Log K_p (skin permeation)	-7.19 cm/s

Druglikeness	
Lipinski	Yes; 0 violation
Ghose	Yes
Veber	Yes
Egan	Yes
Muegge	Yes
Bioavailability Score	0.55

Medicinal Chemistry	
PAINS	0 alert
Brenk	0 alert
Leadlikeness	Yes
Synthetic accessibility	4.62

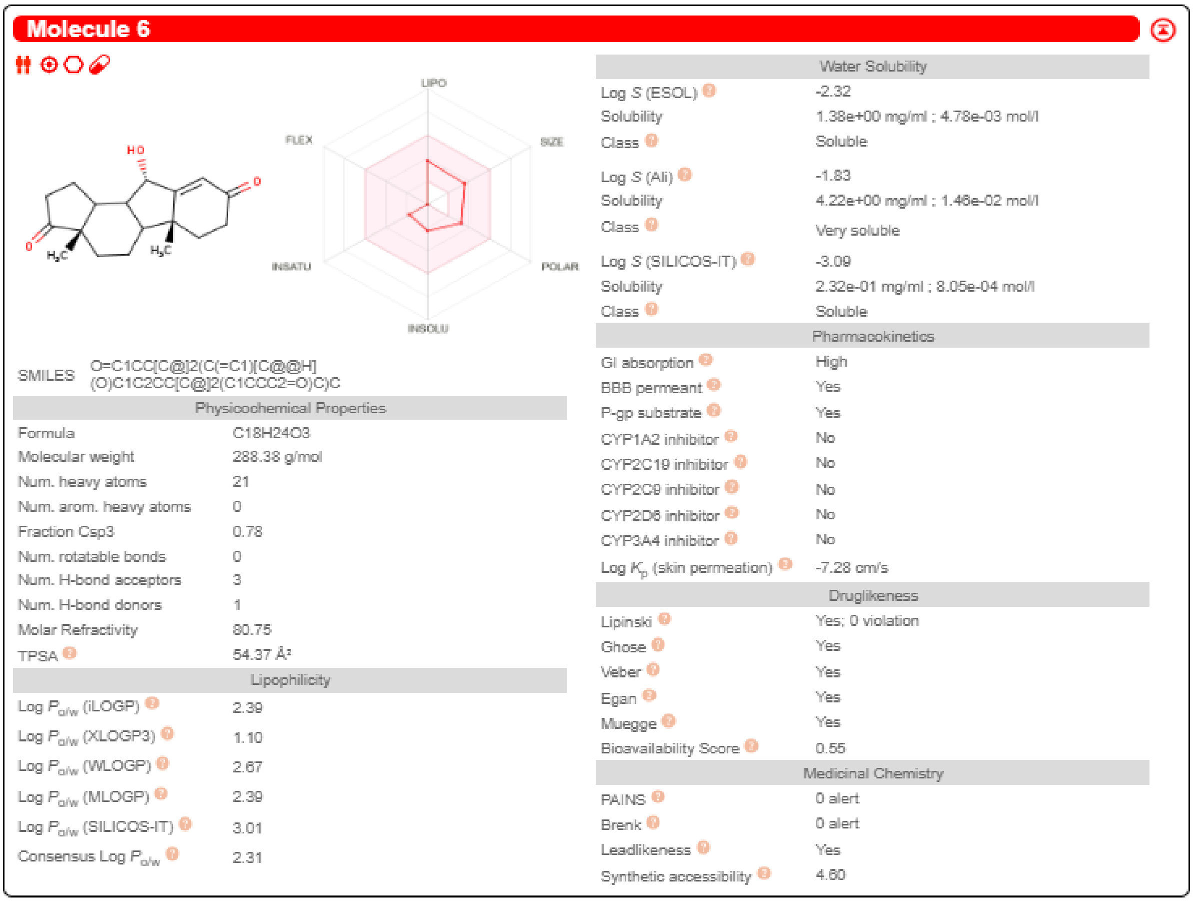

Molecule 6

SMILES O=C1CC[C@]2(C(=C1)[C@@H](O)C1C2CC[C@]2(C1CCC2=O)C)C

Physicochemical Properties	
Formula	C18H24O3
Molecular weight	288.38 g/mol
Num. heavy atoms	21
Num. arom. heavy atoms	0
Fraction Csp3	0.78
Num. rotatable bonds	0
Num. H-bond acceptors	3
Num. H-bond donors	1
Molar Refractivity	80.75
TPSA	54.37 Å²

Lipophilicity	
Log $P_{o/w}$ (iLOGP)	2.39
Log $P_{o/w}$ (XLOGP3)	1.10
Log $P_{o/w}$ (WLOGP)	2.67
Log $P_{o/w}$ (MLOGP)	2.39
Log $P_{o/w}$ (SILICOS-IT)	3.01
Consensus Log $P_{o/w}$	2.31

Water Solubility	
Log *S* (ESOL)	-2.32
Solubility	1.38e+00 mg/ml ; 4.78e-03 mol/l
Class	Soluble
Log *S* (Ali)	-1.83
Solubility	4.22e+00 mg/ml ; 1.46e-02 mol/l
Class	Very soluble
Log *S* (SILICOS-IT)	-3.09
Solubility	2.32e-01 mg/ml ; 8.05e-04 mol/l
Class	Soluble

Pharmacokinetics	
GI absorption	High
BBB permeant	Yes
P-gp substrate	Yes
CYP1A2 inhibitor	No
CYP2C19 inhibitor	No
CYP2C9 inhibitor	No
CYP2D6 inhibitor	No
CYP3A4 inhibitor	No
Log K_p (skin permeation)	-7.28 cm/s

Druglikeness	
Lipinski	Yes; 0 violation
Ghose	Yes
Veber	Yes
Egan	Yes
Muegge	Yes
Bioavailability Score	0.55

Medicinal Chemistry	
PAINS	0 alert
Brenk	0 alert
Leadlikeness	Yes
Synthetic accessibility	4.60

Figure S23. *In silico* physicochemical properties (ADME profile) of hydroxy metabolites (**4-6**)

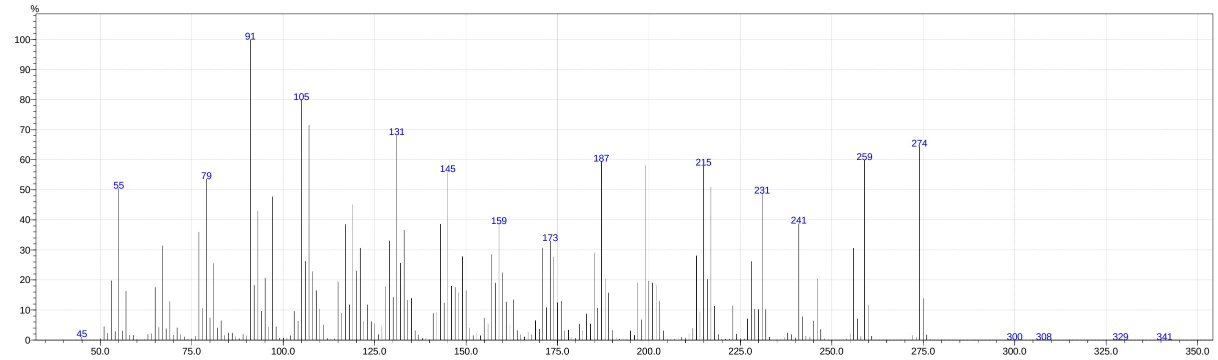

Figure S24. MS spectrum of 3β-hydroxy-B-norandrost-5-en-17-one (**1**).

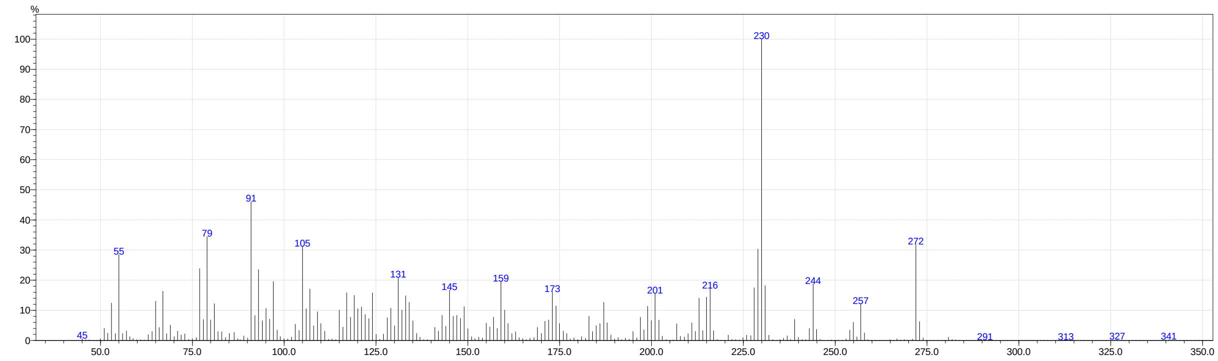

Figure S25. MS spectrum of B-norandrost-4-en-3,17-dione (**3**).

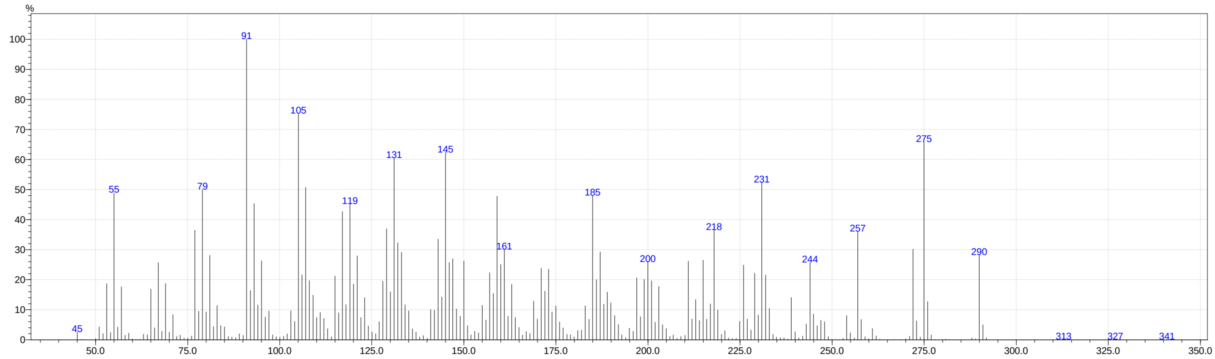

Figure S26. MS spectrum of 3β,15α-dihydroxy-B-norandrost-5-en-17-one (**4**).

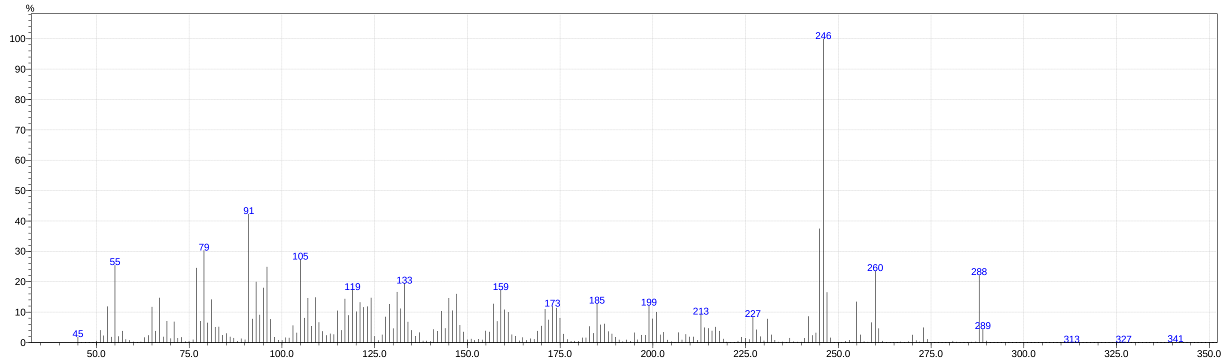

Figure S27. MS spectrum of 15α-hydroxy-B-norandrost-4-en-3,17-dione (**5**).

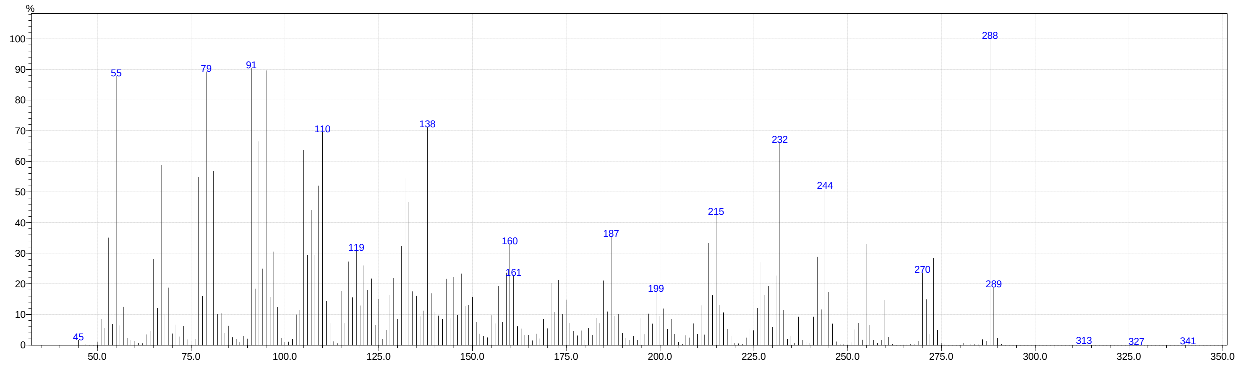

Figure S28. MS spectrum of 6α-hydroxy-B-norandrost-4-en-3,17-dione (**6**).

Table S1. Composition of crude mixtures obtained in transformations of 3β-acetoxy-B-norandrost-5-en-17-one (**2**) by *F. culmorum* AM282.

**Compounds present in the mixture (%)***	**Time of transformation [h]**		
	**9**	**24**	**30**
3β-acetoxy-B-norandrost-5-en-17-one (**2**)	84,13±1,81	21,15±2,44	19,22±2,19
3β-hydroxy-B-norandrost-5-en-17-one (**1**)	8,78±1,15	3,97±0,62	43,89±0,52
3β,15α-dihydroxy-B-norandrost-5-en-17-one (**4**)	7,08±0,68	71,43±2,86	73,26±1,38

*Determined by GC analysis. The presented mean values and standard deviations are measurements of three independent experiments.

Table S2. Comparison of the composition of the product mixtures obtained in transformations of B-nor-DHEA (**1**) and its natural analogue DHEA by *F. culmorum* AM282.

**Substrate**	**Compounds present in the mixture (%)***	**Time of transformation [h]**			
		**3**	**6**	**9**	**12**
B-nor-DHEA (**1**)	B-nor-DHEA (**1**)	98.97±0.34	88.57±1.64	55.07±2.96	8.30±1.79
	15α-hydroxy-B-nor-DHEA (**4**)	1.03±0.34	11.42±1.64	44.93±2.96	91.7±1.79
DHEA	DHEA	96.27±1.19	79.57±4.86	24.60±3.45	2.02±0.62
	7α-hydroxy-DHEA	3.73±1.19	20.43±4.85	75.40±3.45	97.98±0.62
equimolar mixture of DHEA and B-nor-DHEA (**1**)	B-nor-DHEA (**1**)	49.67±0.19	41.93±1.48	18.70±1.79	5.13±1.89
	15α-hydroxy-B-nor-DHEA (**4**)	0.33±0.12	8.10±1.59	33.87±2.10	44.87±2.04
	DHEA	49.10±0.16	37.60±1.16	11.11±0.98	1.89±0.49
	7α-hydroxy-DHEA	0.90±0.22	12.37±0.90	39.32±1.23	48.11±0.35

*Determined by GC analysis. The presented values are the average of three independent experiments and relative differences between the border values have not exceeded 5%.

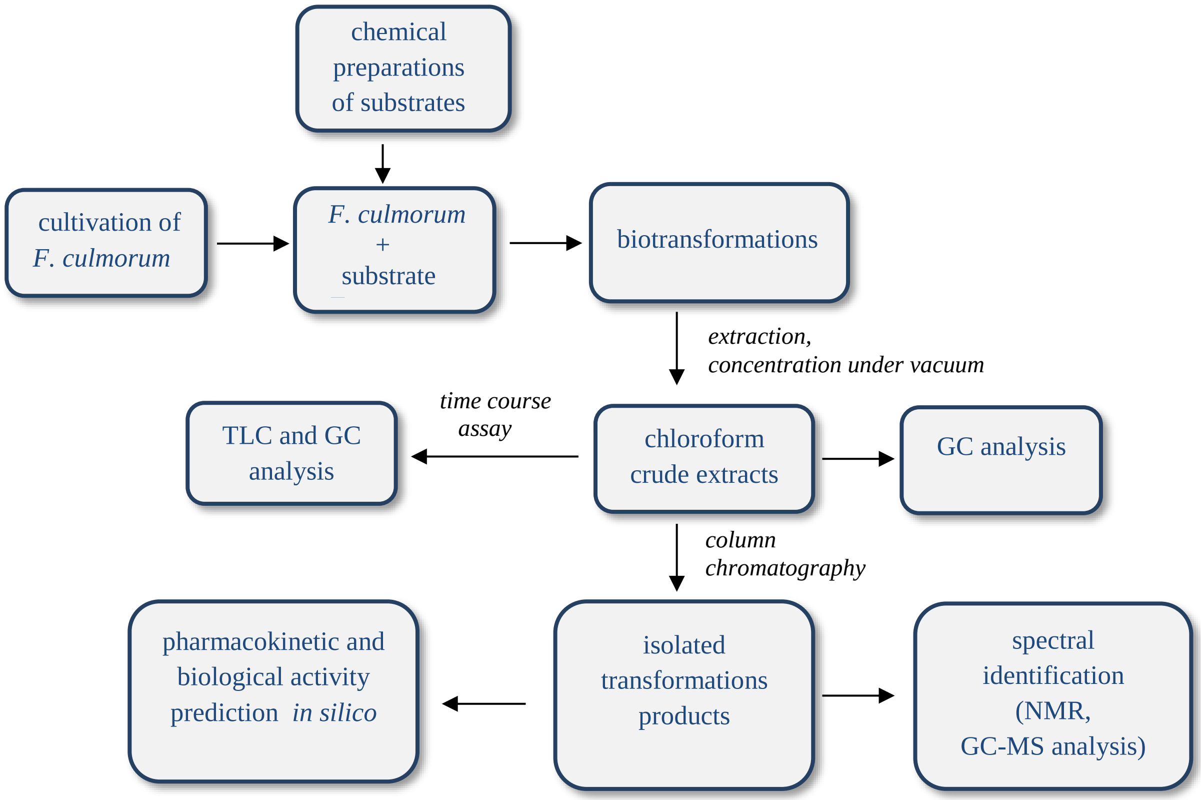

Scheme S1. Flowchart depicting the main stages of the studies.
